# The virome of the invasive Asian bush mosquito *Aedes japonicus* in Europe

**DOI:** 10.1093/ve/vead041

**Published:** 2023-07-03

**Authors:** Sandra R Abbo, João P P de Almeida, Roenick P Olmo, Carlijn Balvers, Jet S Griep, Charlotte Linthout, Constantianus J M Koenraadt, Bruno M Silva, Jelke J Fros, Eric R G R Aguiar, Eric Marois, Gorben P Pijlman, João T Marques

**Affiliations:** Laboratory of Virology, Wageningen University & Research, Droevendaalsesteeg 4, Wageningen 6708 PB, The Netherlands; Department of Biochemistry and Immunology, Instituto de Ciências Biológicas, Universidade Federal de Minas Gerais, Av. Antonio Carlos 6627, Belo Horizonte 31270-901, Brazil; Insect Models of Innate Immunity, Université de Strasbourg, CNRS UPR9022, INSERM U1257, 2 Allee Konrad Roentgen, Strasbourg 67000, France; Laboratory of Virology, Wageningen University & Research, Droevendaalsesteeg 4, Wageningen 6708 PB, The Netherlands; Laboratory of Entomology, Wageningen University & Research, Droevendaalsesteeg 4, Wageningen 6708 PB, The Netherlands; Laboratory of Virology, Wageningen University & Research, Droevendaalsesteeg 4, Wageningen 6708 PB, The Netherlands; Laboratory of Entomology, Wageningen University & Research, Droevendaalsesteeg 4, Wageningen 6708 PB, The Netherlands; Laboratory of Entomology, Wageningen University & Research, Droevendaalsesteeg 4, Wageningen 6708 PB, The Netherlands; Laboratory of Entomology, Wageningen University & Research, Droevendaalsesteeg 4, Wageningen 6708 PB, The Netherlands; Department of Biochemistry and Immunology, Instituto de Ciências Biológicas, Universidade Federal de Minas Gerais, Av. Antonio Carlos 6627, Belo Horizonte 31270-901, Brazil; Laboratory of Virology, Wageningen University & Research, Droevendaalsesteeg 4, Wageningen 6708 PB, The Netherlands; Department of Biochemistry and Immunology, Instituto de Ciências Biológicas, Universidade Federal de Minas Gerais, Av. Antonio Carlos 6627, Belo Horizonte 31270-901, Brazil; Department of Biological Science, Center of Biotechnology and Genetics, State University of Santa Cruz, Rod. Jorge Amado Km 16, Ilhéus 45662-900, Brazil; Insect Models of Innate Immunity, Université de Strasbourg, CNRS UPR9022, INSERM U1257, 2 Allee Konrad Roentgen, Strasbourg 67000, France; Laboratory of Virology, Wageningen University & Research, Droevendaalsesteeg 4, Wageningen 6708 PB, The Netherlands; Department of Biochemistry and Immunology, Instituto de Ciências Biológicas, Universidade Federal de Minas Gerais, Av. Antonio Carlos 6627, Belo Horizonte 31270-901, Brazil; Insect Models of Innate Immunity, Université de Strasbourg, CNRS UPR9022, INSERM U1257, 2 Allee Konrad Roentgen, Strasbourg 67000, France

**Keywords:** virome, *Aedes japonicus*, mosquito, RNA interference, metagenomics, narnavirus, totivirus, anphevirus, bunyavirus, rhabdovirus

## Abstract

The Asian bush mosquito *Aedes japonicus* is rapidly invading North America and Europe. Due to its potential to transmit multiple pathogenic arthropod–borne (arbo)viruses including Zika virus, West Nile virus, and chikungunya virus, it is important to understand the biology of this vector mosquito in more detail. In addition to arboviruses, mosquitoes can also carry insect-specific viruses that are receiving increasing attention due to their potential effects on host physiology and arbovirus transmission. In this study, we characterized the collection of viruses, referred to as the virome, circulating in *Ae. japonicus* populations in the Netherlands and France. Applying a small RNA-based metagenomic approach to *Ae. japonicus*, we uncovered a distinct group of viruses present in samples from both the Netherlands and France. These included one known virus, *Ae. japonicus* narnavirus 1 (AejapNV1), and three new virus species that we named *Ae. japonicus* totivirus 1 (AejapTV1), *Ae. japonicus* anphevirus 1 (AejapAV1) and *Ae. japonicus* bunyavirus 1 (AejapBV1). We also discovered sequences that were presumably derived from two additional novel viruses: *Ae. japonicus* bunyavirus 2 (AejapBV2) and *Ae. japonicus* rhabdovirus 1 (AejapRV1). All six viruses induced strong RNA interference responses, including the production of twenty-one nucleotide-sized small interfering RNAs, a signature of active replication in the host. Notably, AejapBV1 and AejapBV2 belong to different viral families; however, no RNA-dependent RNA polymerase sequence has been found for AejapBV2. Intriguingly, our small RNA-based approach identified an ∼1-kb long ambigrammatic RNA that is associated with AejapNV1 as a secondary segment but showed no similarity to any sequence in public databases. We confirmed the presence of AejapNV1 primary and secondary segments, AejapTV1, AejapAV1, and AejapBV1 by reverse transcriptase polymerase chain reaction (PCR) in wild-caught *Ae. japonicus* mosquitoes. AejapNV1 and AejapTV1 were found at high prevalence (87–100 per cent) in adult females, adult males, and larvae. Using a small RNA-based, sequence-independent metagenomic strategy, we uncovered a conserved and prevalent virome among *Ae. japonicus* mosquito populations. The high prevalence of AejapNV1 and AejapTV1 across all tested mosquito life stages suggests that these viruses are intimately associated with *Ae. japonicus*.

## Background

Mosquitoes of the *Aedes* genus are responsible for mosquito-borne viral disease outbreaks worldwide. The tropical yellow fever mosquito *Aedes aegypti* and the invasive Asian tiger mosquito *Aedes albopictus* pose a large threat to human health by transmitting medically important arthropod-borne (arbo)viruses including Zika virus (ZIKV), chikungunya virus (CHIKV), dengue virus, and yellow fever virus (Kraemer et al. 2019; Souza-Neto, Powell, and Bonizzoni 2019). Research has especially focused on these two urban mosquito species; however, also other *Aedes* species are becoming increasingly widespread (Montarsi et al. 2013; Janssen et al. 2020; Pagac et al. 2021), and their role in arbovirus transmission requires further attention.

The Asian bush mosquito *Aedes japonicus*, which originates in Northeast Asia, is a highly invasive species (Kampen and Werner 2014). During the past two decades, this mosquito species quickly spread to North America and Europe, where it established large, permanent populations despite intensive control efforts (Kampen and Werner 2014; Ibanez-Justicia et al. 2018). Importantly, *Ae. japonicus* is capable of transmitting multiple arboviruses including West Nile virus (Turell et al. 2001; Veronesi et al. 2018), Japanese encephalitis virus (Takashima and Rosen 1989), ZIKV (Jansen et al. 2018; Abbo et al. 2020), Usutu virus (USUV) (Abbo et al. 2020), and CHIKV (Schaffner et al. 2011). Therefore, it is important to understand the biology of this exotic mosquito species in greater detail.

In addition to arboviruses, mosquitoes can also carry insect-specific viruses (ISVs). Although arboviruses are maintained in transmission cycles between arthropods and vertebrate animals or humans, ISVs do not replicate in vertebrates and only infect insects. ISVs can persistently infect mosquito populations and have recently received increasing attention due to their potential effects on host physiology and arbovirus transmission (Romo et al. 2018; Agboli et al. 2019; Baidaliuk et al. 2019; de Almeida et al. 2021; Olmo et al. 2023). Exploring the diversity of ISVs may also help to better understand the evolution of arboviruses and to develop new strategies for arbovirus control (Bolling et al. 2015).

Extensive virome analyses that have been performed for *A. aegypti* and *A. albopictus* (Zakrzewski et al. 2018; Shi et al. 2019, 2020; Olmo et al. 2023) showed that both mosquito species harbour a large diversity of ISVs. For *Ae. japonicus*, however, an in-depth virome analysis is still missing, although a first glimpse has revealed the presence of a novel narnavirus in this mosquito. *Aedes japonicus* narnavirus 1 (AejapNV1) was discovered in *Ae. japonicus* from the Netherlands (Abbo et al. 2020) and was also found in *Ae. japonicus* from Japan shortly thereafter (Faizah et al. 2020), indicating its close association with *Ae. japonicus*. AejapNV1 belongs to a novel group of unique, ambigrammatic narnaviruses, which not only contain an open reading frame (ORF) coding for the RNA-dependent RNA polymerase (RdRp) on the positive strand but also a very long ORF with unknown function on the (reverse complement) negative strand (DeRisi et al. 2019; Dinan et al. 2020).

Metagenomic approaches have played an essential role in uncovering the virome of vector mosquitoes and the discovery of many novel ISVs (de Almeida et al. 2021). Despite the success of these approaches, certain aspects of virome analysis remain challenging to fulfil using large-scale nucleic acid sequencing, such as the detection and classification of highly divergent viral sequences that do not align to any known reference sequence (i.e. the viral ‘dark matter’), the association of sequences from different genomic segments of the same virus, and the differentiation of exogenous viruses from endogenous viral elements (EVEs).

These limitations can be overcome using small RNA-based metagenomics. Virus-derived small RNAs are produced in mosquitoes when viral double-stranded RNA (dsRNA) replication intermediates are recognized and cleaved by Dicer-2, a key protein of the antiviral RNA interference (RNAi) machinery, which results in the production of twenty-one nucleotide (nt) small interfering RNAs (siRNAs) (Aguiar, Olmo, and Marques 2016). The formation of viral siRNAs is a signature of active virus replication (Wu et al. 2010). In addition to viral siRNAs, viral P-element induced wimpy testis (PIWI)–interacting RNAs (piRNAs) of ∼24-29 nt in length are also produced for some viruses (Aguiar, Olmo, and Marques 2016). Thus, small RNA sequencing detects products derived from host antiviral pathways that discriminate EVEs and allow for sequence-independent characterization of viral sequences (Aguiar et al. 2015).

In the current study, we applied a small RNA-based metagenomic approach to analyse the virome of wild-caught *Ae. japonicus* mosquitoes from populations in the Netherlands and France and identified AejapNV1 and three novel virus species. Interestingly, for AejapNV1, not only the primary genome segment (S1) encoding the RdRp was found, but we also discovered an ambigrammatic secondary genome segment (S2) with unknown function. S2 did not align to any known sequence in public databases. Still, it could be associated with the same virus on the basis of similar (di)nucleotide composition in S1 and S2 and by applying unsupervised clustering techniques using small RNA features generated by our small RNA metagenomic approach. Additionally, we found sequences derived from two putative, novel virus species, and together with the other four discovered viruses, we characterized their genome organisation and small RNA profiles. Finally, reverse transcriptase PCR (RT-PCR) was used to study the prevalence of these viruses in *Ae. japonicus* adults, larvae, and eggs.

## Methods

### Small RNA library preparation and high-throughput sequencing

**Figure 1.**
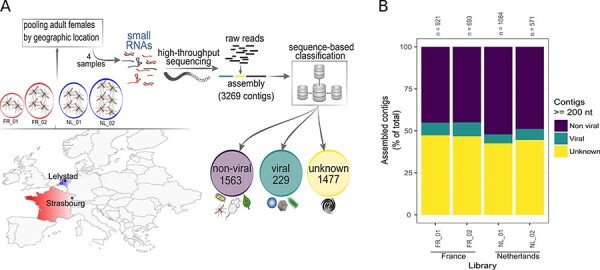
Analysis of the *Ae. japonicus* virome using a small RNA-based metagenomic approach. (A) Map of Europe indicating mosquito collection sites: Strasbourg, France and Lelystad, the Netherlands . Pools with the number of sampled mosquitoes from Strasbourg are indicated inside the circles FR_01, two mosquitoes and FR_02, four mosquitoes; and from Lelystad inside the circles NL_01, four mosquitoes, and NL_02, six mosquitoes. Captured mosquitoes were morphologically identified by species. Samples were used to prepare small RNA libraries for high-throughput sequencing. Sequencing results were analysed using our metagenomic pipeline. Assembled contigs were classified into non-viral, viral, and unknown sequences based on sequence similarity against reference databases. (B) Individual results from our sequence similarity analysis for each of the four small RNA libraries in this study. The total number of contigs larger than or equal to 200 nt (*n*) and the proportion of non-viral, viral and unknown contigs are shown.

*Aedes japonicus* adult female mosquitoes from Strasbourg, France, were collected using human landing catches (HLCs) (Abbo et al. 2020) and further identified using morphological characteristics. Two pools were made prior to RNA extraction, one containing two (FR_01) individuals and another containing four (FR_02) as shown in Fig. 1A. Total RNA was isolated using TRIzol (Invitrogen) following the manufacturer’s protocol. Briefly, mosquitoes were transferred to 1.5-ml screw-capped tubes containing ceramic beads (1.4 mm in diameter, Omini) and ice-cold TRIzol (Invitrogen). Mosquitoes were ground using a Precellys Evolution Homogenizer at 6,500 rpm in three cycles of 20 s each. Then, 10 µg of glycogen (Ambion) was added to the aqueous layer of each sample to facilitate pellet visualization upon RNA precipitation. RNAs were resuspended in RNAse-free water (Ambion) and stored at −80°C until further notice. Total RNA was used as input for library preparation utilizing the kit NEBnext Multiplex Small RNA Library Prep Set for Illumina following the recommended protocol, except for one minor modification: the 5ʹ adapter was replaced by an analogue that contains six extra nucleotides at the 3ʹ extremity to improve barcoding precision, which is sequenced along with the cloned small RNA. Libraries were sequenced at the GenomEast sequencing platform at Institut de Génétique et de Biologie Moléculaire et Cellulaire in Strasbourg, France, using Illumina HiSeq 4000 equipment. The *Ae. japonicus* small RNA libraries from Lelystad, the Netherlands, reanalysed in this study, were previously deposited in the National Center for Biotechnology Information (NCBI) Short Read Archive (SRA) under BioProject PRJNA545039 (Abbo et al. 2020). Libraries NL_01 and NL_02 were built from pools of four and six adult females, respectively (Fig. 1A). Note that depletion of ribosomal RNAs from total RNA was not necessary since the library construction strategy is focused on small RNAs (Aguiar et al. 2015).

### Small RNA-based metagenomics for virus identification

Raw reads from small RNA libraries were submitted to adapter trimming using Cutadapt v1.12 (Martin 2011), and Illumina libraries from France had the six inserted nucleotides trimmed with the adapters. Sequences with an average Phred quality below 20, ambiguous nucleotides, and/or a length shorter than 15 nt were discarded. The remaining sequences were mapped to genome sequences of *A. aegypti* (AaeL5) (Matthews et al. 2018), *A. albopictus* (Chen et al. 2015), and bacterial reference genomes using Bowtie v1.3 (Langmead et al. 2009). Unmapped reads from the previous step were assembled in contigs combining metaSPAdes (Nurk et al. 2017) and Velvet v1.0.13 (Zerbino and Birney 2008). More details about k-mers and read sizes combinations for assemblies using small RNAs in the current work can be found in Aguiar et al. (2015). Contigs larger than 200 nt were characterized based on sequence similarity against the NCBI *nt* database using BLAST+ (Camacho et al. 2009) and against *nr* using DIAMOND (mode --*very sensitive*) (Buchfink, Reuter, and Drost 2021), considering significant hits with *e-values* lower than 1 × 10^−5^ for nucleotide comparison or 1 × 10^−3^ for amino acid comparison. For small RNA size profile and coverage analysis, reads unmapped to mosquito and bacterial genomes were aligned to viral and unknown contigs using Bowtie v1.3, allowing one mismatch. Size profiles of small RNAs matching reference sequences and 5ʹ nt base preference were calculated using in-house Perl v5.16.3, BioPerl library v1.6.924, and R v4.0.5 scripts (https://github.com/JPbio/small_RNA_MetaVir/). Plots were generated in R using ggplot2 v3.3.5 package. For manual curation of putative viral contigs, top five BLAST and DIAMOND hits were analysed to rule out the similarity to other organisms; ORF organization and small RNA profiles (size distribution and coverage) were analysed to differentiate exogenous from endogenous viruses and check for inconsistencies. Contigs containing truncated ORFs and small RNA profiles without symmetric small RNA peaks at 21 nt were considered putative EVEs and removed from further analysis as described before (Aguiar, Olmo, and Marques 2016; Olmo et al. 2023). Curated viral contigs were clustered by sequence similarity using CD-HIT (Fu et al. 2012) requiring 90 per cent coverage of the smaller sequence (*-aS*) with 90 per cent of global identity (*-c*) to remove redundancy. Representative unique contigs from the CD-HIT clustering were used for co-occurrence analysis based on small RNA abundancy in each of the small RNA libraries. A dendogram was generated based on hierarchical clustering of each representative unique contig based on Reads Per Kilobase of transcript per Million reads (RPKM) values of 20 to 22 nt small RNAs aligned to the contigs in each of the four independent libraries. The dendogram was generated in R using Euclidean distance and average method. For small RNA profiles, *Z*-scores were calculated based on the frequency of each small RNA size from 15 to 35 nt mapped to representative contig considering each strand polarity separately using the library where the contig was originally assembled. Strand polarity for contig comparisons was adjusted based on the reads abundance per strand and coding ORFs direction of each contig. Heatmaps for RPKM and *Z*-score values were plotted using the package ComplexHeatmap in R (Gu, Eils, and Schlesner 2016). As an attempt at viral sequence extension as proposed by Sardi et al. (2020), contigs grouped in the same cluster with sequence similarity hits to the same virus were submitted to a new assembly round using SPAdes (Bankevich et al. 2012) with the parameter --*trusted-contigs* using all the libraries in which that viral sequences were found.

### piRNA analysis

Sequence overlaps and signatures of viral-derived small RNAs were analysed to determine the presence of piRNAs. Distances between small RNAs were calculated as previously described (Han et al. 2015; Gainetdinov et al. 2018). Briefly, the frequency of 5′–5′ distances between 24-29 nt long reads from opposite strands was calculated and normalized by *Z*-score. A–U enrichment for first and tenth positions of 24-29 nt long reads was observed by filtering these reads after alignment to viral sequences and plotting sequence logos using the ggseqlogo R package (Wagih 2017).

### Phylogenetic analysis

We selected the contig containing the largest RdRp gene sequence for the identified viruses. For bunyaviruses, we also selected the contigs containing the largest sequences of nucleocapsid and glycoprotein genes. The presence of conserved protein domains for RdRp, nucleocapsid, and glycoprotein was confirmed with NCBI Conserved Domain Search (https://www.ncbi.nlm.nih.gov/Structure/cdd/wrpsb.cgi). Searches for potential homologous viral sequences were performed using Blastx against the *nr* database. Coding sequences were translated to amino acids, and multiple sequence alignments were performed using the software **m**ultiple **a**lignment using **f**ast **F**ourier **t**ransform (Katoh et al. 2002). The best-fit protein evolution model was selected using MEGA-X (Kumar et al. 2018) under the Akaike Information Criterion. Phylogenetic inference was executed with MEGA-X using the maximum likelihood method. For all phylogenetic trees, clade robustness was assessed using the bootstrap method (1,000 pseudoreplicates). The trees were viewed using iTOL version 6.5 (Letunic and Bork 2021).

### Analyses of CpG dinucleotide frequency and GC content

Mononucleotide frequencies, dinucleotide frequencies, and the observed/expected (O/E) ratios of viral and unknown sequences were determined using the function Composition Scan within SSE version 1.4 (Simmonds 2012).

### Sequence alignment and RNA structure modelling

Narnavirus RNA sequences were aligned using MUSCLE version 3.8.1551 (Edgar 2004). Narnavirus RNA secondary structures were predicted using the RNAstructure web server version 6.3 with the temperature set to 28°C (Reuter and Mathews 2010). Pseudoknots in totivirus RNA were predicted using DotKnot version 1.3.2 (Sperschneider and Datta 2010). RNA folding was visualised using VARNA (Darty, Denise, and Ponty 2009).

### Protein structure analysis

Primary structure physical–chemical properties were determined using ProtParam software (Wilkins et al. 1999). Global sequence alignments were performed using Clustal Omega (Sievers et al. 2011) and coloured using ESPcript web server (Robert and Gouet 2014). PSIPRED Workbench (Buchan et al. 2013) was used for structural assessments, with secondary structures predicted using PSIPRED 4.0 (Buchan and Jones 2019), DISOPRED3 (Jones and Cozzetto 2015) for disordered regions, and MEMSAT-SVM (Nugent and Jones 2009) for membrane interaction. AlphaFold (Jumper et al. 2021) tertiary structure modelling was performed locally with the complete database downloaded and using default parameters and ‘6 January 2022’ as the maximum template date.

### Mosquito collection and rearing for RT-PCR

To obtain samples for RT-PCR analyses, *Ae. japonicus* mosquitoes were collected in Lelystad, the Netherlands using oviposition traps, water reservoirs in local rain barrels and HLCs as described (Abbo et al. 2020) during the summer of 2020. Adult females obtained from HLCs were stored at −80°C until further analysis, whereas eggs and larvae were kept in the laboratory at 23°C and a relative humidity of 60 per cent. Adult males were grown from collected eggs and larvae as described (Abbo et al. 2020) and subsequently stored at −80°C. Pools of twenty-five eggs or individual fourth instar larvae were also stored at −80°C. The common house mosquito *Culex pipiens* (biotype *pipiens*) was used as negative control for the RT-PCR analyses. *Culex pipiens* mosquitoes collected in Wageningen, the Netherlands, during the summer of 2020, were used to establish a laboratory colony as previously described (Vogels et al. 2016). Mosquitoes were fed with chicken whole blood (Kemperkip, Uden, the Netherlands) through Parafilm using a Hemotek PS5 feeder (Discovery Workshops, Lancashire, UK) and reared at 23°C and a relative humidity of 60 per cent. Individual adult female mosquitoes were stored at −80°C for further analysis.

### Mosquito RNA isolation for RT-PCR

Frozen mosquitoes were homogenized using a Bullet Blender Storm (Next Advance, Averill Park, NY, USA) in combination with 0.5 mm zirconium oxide beads (Next Advance; for adult mosquitoes and larvae) or 0.9–2.0 mm stainless steel beads (Next Advance; for eggs) at maximum speed for 2 min. Afterwards, homogenates were centrifuged at maximum speed in an Eppendorf 5424 centrifuge for 1 min. Pellets were resuspended in 450 μl lysis buffer from the innuPREP DNA/RNA Mini Kit (Analytik Jena, Jena, Germany) and subsequently incubated for 20 min to lyse. RNA was isolated using the innuPREP DNA/RNA Mini Kit (Analytik Jena) according to the manufacturer’s instructions. RNA yields were measured using a NanoDrop ND-1000 spectrophotometer.

### RT-PCR analysis

Viruses were detected by RT-PCR using a 2720 Thermal Cycler (Applied Biosystems, Foster City, CA, USA) and SuperScript III One-Step RT-PCR System with Platinum *Taq* DNA polymerase (Invitrogen) according to the manufacturer’s protocol. In the case of a dsRNA virus, total RNA was subjected to a 5-min incubation step at 95°C prior to the RT-PCR reaction to denature the viral dsRNA. Mosquito total RNA (50 ng) was used as input for RT-PCR, and each virus was amplified using specific primers targeting the viral RdRp sequence (Table 1). For AejapNV1, primers were not only designed for the primary segment (RdRp) but also for the secondary segment (Table 1). To test the RNA quality of the samples, RT-PCRs against mosquito ribosomal protein S7 (RPS7) RNA were also included (Table 1). The RT-PCR product of the secondary segment of AejapNV1 was sent for DNA sequencing to Macrogen (EZ-Seq protocol).

**Table 1.** Primer sets used in this study.

Target	Primer name	Primer sequence (5ʹ → 3ʹ)	Product (bp)
AejapNV1 primary segment	AejapNV1 S1 FW	TCGAGGTGACGACCTGGTTG	1060
	AejapNV1 S1 RV	CTTGGCCTTGACGGTCAGCT	
AejapNV1 secondary segment	AejapNV1 S2 FW	CCTTCCGTGGAGAATACTGG	774
	AejapNV1 S2 RV	TTTGGACGGTGATACCACGG	
AejapTV1	AejapTV1 FW	CCAATAGTGAACGCCTGGTC	998
	AejapTV1 RV	GGTGCTTCCAGATGAACACC	
AejapAV1	AejapAV1 FW	ACGCCATGCTTGCTCTAATC	1043
	AejapAV1 RV	AGCAAGGTAGGAGTCGAAGG	
AejapBV1	AejapBV1 FW	ACCTTCCAGAGAGCATGGAG	1095
	AejapBV1 RV	TAGCATGGGTAGATTGCAGC	
RPS7	RPS7 FW	ATGGTTTTCGGATCAAAGGT	175
	RPS7 RV	CGATAGCCTTCTTGCTGTTG	

## Results

### Classification of contigs based on sequence similarity

To assess the collection of viruses found in *Ae. japonicus*, two new small RNA libraries of *Ae. japonicus* from France (FR_01 and FR_02) were sequenced and two published small RNA libraries of *Ae. japonicus* from the Netherlands (NL_01 and NL_02) (Abbo et al. 2020) were reanalysed (Fig. 1A; Supplementary Table S1). Libraries were processed and analysed following a small RNA-based metagenomic strategy optimized to detect viral sequences (Olmo et al. 2023). General information on small RNA libraries and SRA accessions can be found in Supplementary Table S1.

In total, 3,269 contiguous sequences (contigs) larger than 200 nt were assembled from the four individual libraries. A summary of the assembly results is shown in Supplementary Table S2. Based solely on sequence similarity searches against NCBI non-redundant nucleotide and protein databases (*nt* and *nr*), contigs were classified into 229 viral, 1,563 non-viral, and 1,477 unknown sequences (Fig. 1A; Supplementary Table S3). Proportions of each class per library are shown in Fig. 1B.

### Curation of viral contigs

EVEs are often misperceived as viruses in metagenomic studies based on RNA sequencing (also referred to as metatranscriptomics), and this can overestimate the number of viruses in a given sample. To discriminate sequences derived from viruses and EVEs, we used a previously established analysis pipeline (Olmo et al. 2023). Briefly, this pipeline relies on investigating the small RNA profile and ORF composition of each contig previously classified as of viral origin by sequence similarity. Applying this filter, ninety-three sequences derived from exogenous viruses were obtained (Supplementary Table S3) out of the initial 229 viral contigs (Supplementary Table S4). The remaining ninety-three contigs were clustered by sequence similarity using CD-HIT to remove redundancy, resulting in fifty-six unique representative viral contigs (Supplementary Table S5). To evaluate only the natural circulating virome, contigs from viruses that were experimentally introduced, three USUV and fourteen ZIKV matching sequences, assembled in the libraries NL_01 and NL_02 were removed, leaving a total of thirty-nine unique representative sequences.

**Figure 2.**
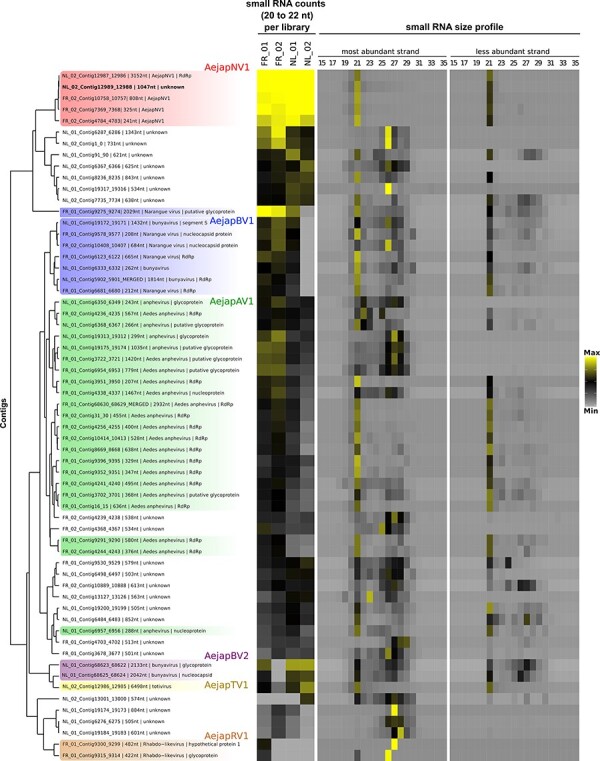
Co-occurrence of viral and unknown contigs. Hierarchical clustering of viral and unknown contigs assembled from small RNAs derived from *Ae. japonicus*. Clustering was based on the Euclidean distance of RPKM values of small RNA counts with size from 20 to 22 nt in each library applying average method. Contig clusters were defined using the dendrogram. Contigs inferred to be from the same virus were coloured equally. Heatmap on the left represents the small RNA abundance for each curated contig in *Ae. japonicus* libraries, but this was not considered for the hierarchical clustering. We plotted the Log2 of the RPKM values of small RNA counts with sizes from 20 to 22 nt (maximum value: 10; minimum value: 0). Heatmap on the right represents *Z*-score values for small RNAs from each size from 15 to 35 nt divided by strand polarity in the library in which the contig was originally assembled (maximum value: 7; minimum value: −1).

In an effort to associate different segments that may belong to the same virus, we evaluated co-occurrence of the viral contigs based on small RNA counts in the four libraries as previously reported (Olmo et al. 2023). In this regard, we determined the normalized number of small RNA reads that could be mapped to each of the thirty-nine representative viral contigs. Here, we also included twenty-two non-redundant contigs of unknown origin, which have lengths larger than 500 nt and passed the filters based on the small RNA profile and ORF composition similar to viral sequences. This analysis identified several clusters of co-occurring contigs (Fig. 2). Co-occurring contigs were considered probable fragments from the same virus, especially when they shared a similar small RNA size profile (Fig. 2). In addition, contigs that showed significant similarity to the same reference in the database were also considered part of the same virus (Supplementary Table S4). Based on these two criteria, co-occurrence and closest reference in the database, we defined five groups of viral contigs.

The first group, highlighted in red, formed a single cluster containing four contigs with high sequence similarity to AejapNV1 and an additional unknown contig (NL_02_Contig12989_12988) (Fig. 2). The second group, highlighted in blue, includes contigs with significant similarity to different segments of Narangue virus, a bunyavirus, mostly within a cluster except for one contig with high similarity to a bunyavirus glycoprotein (FR_01_Contig9275_9274) (Fig. 2). Another large group of sequences, highlighted in green, were divided into two major clusters and displayed significant similarity to *Aedes anphevirus* (Fig. 2). Two other groups, one containing two contigs with significant sequence similarity to a bunyavirus (highlighted in purple) and one contig similar to a totivirus (highlighted in yellow) were found in a common cluster (Fig. 2). Finally, two contigs with high sequence similarity to a rhabdovirus also formed a clear group in single cluster (highlighted in brown in Fig. 2). Other clusters were not highlighted because they were composed of unknown contigs that could not clearly be associated with a specific virus. Apart from ZIKV and USUV, which were introduced by artificial infection, no contigs showed similarity to known arboviruses.

### Virus identification

**Figure 3.**
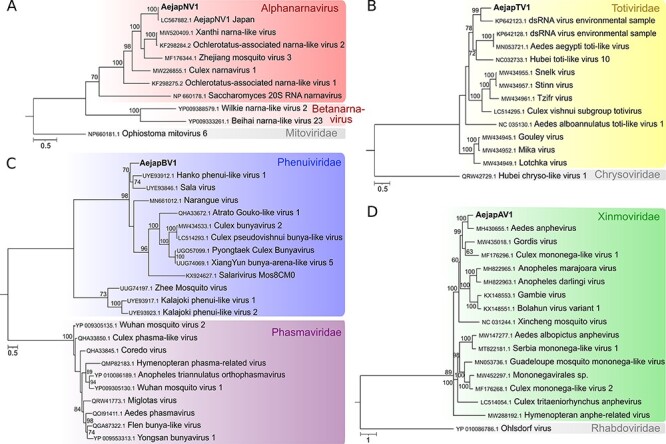
Phylogeny of viruses identified in *Ae. japonicus* mosquitoes. Phylogenetic trees were generated using the multiple sequence alignments of RdRp amino acid sequences. The trees were inferred by using the maximum likelihood method. The tree with the highest log likelihood is shown for each virus. Number of conserved sites and the substitution models used for each tree: (A) AejapNV1, 1446 sites, LG + G + F; (B) AejapTV1, 1269 sites, LG + G; (C) AejapBV1, 616 sites, LG + G + F; and (D) AejapAV1, 1188 sites, LG + G + I + F. Node bootstraps were calculated with 1,000 replicates and are shown close to each clade, and values < 60 were omitted. Trees were midpoint-rooted, and RdRp sequences from distinct viral families were included in the alignments as outgroups. The trees are drawn to scale, and branch lengths represent expected numbers of substitutions per amino acid site. Accession numbers for the nucleotide sequences from which the corresponding protein sequences were derived or the direct protein sequences are shown with the virus names. Viruses identified in this study are in bold.

To further characterize putative viruses represented by the contigs from the clusters highlighted in Fig. 2, we focused on sequences encoding viral polymerases. It was possible to identify contigs encoding viral polymerases in four clusters that were compared to the most similar sequences in GenBank (Table 2). One cluster contained AejapNV1 (genus *Narnavirus*, family *Narnaviridae*) that had been previously identified as a possible ISV (Abbo et al. 2020). According to phylogenetic analysis (Fig. 3), this virus clustered with other mosquito-associated narnaviruses containing long reverse ORFs (rORFs) from the *Alphanarnavirus* clade (Dinan et al. 2020).

**Table 2.** Viruses discovered by *de novo* assembly of small RNA reads from *Ae. japonicus* mosquitoes collected in the Netherlands and France.

Virus	Segment	Virus family	Genome	Query size^a^ (nt)	Query coverage (%)	Identity (%)	*E*-value	Type	Closest sequence in GenBank
AejapNV1	1	*Narnaviridae*	+ssRNA	3152	100	100	0.0	nt	MK984721.2
	2			1047	N/A	N/A	N/A	N/A	N/A
AejapTV1	N/A	*Totiviridae*	dsRNA	6498	47	60	0.0	aa	AJT39583.1
AejapAV1	N/A	*Xinmoviridae*	−ssRNA	2932	99	58	0.0	aa	AWW13479.1
AejapBV1	L	*Phenuiviridae*	−ssRNA	1814	96	35	5.0 × 10^−9^	aa	QHA33858.1
	M			2029	85	42	1.0 × 10^−142^		QHA33860.1
	S			1432	55	44	8.0 × 10^−62^		QHA33859.1
bAejapBV2	M	*Phasmaviridae*	−ssRNA	2133	88	44	0	aa	YP_009305132.1
	S			2042	52	54	5.0 × 10^−126^		YP_009305134.1
bAejapRV1	N/A	Unknown	−ssRNA	482	57	38	2.0 × 10^−8^	aa	QIS62330.1

a The length in nucleotides of the largest assembled contig.

b Putative novel virus species, for which we were unable to detect RdRp sequences.

The other contigs containing viral polymerase sequences showed significant but low sequence similarity to known references of totiviruses, anpheviruses, and bunyaviruses in GenBank only at the amino acid level (*e*-value < 1 × 10^−3^) (Table 2). Phylogenetic analyses indicated that these are likely new viruses belonging to the *Totiviridae, Xinmoviridae*, and *Phenuiviridae* families that were named *Ae. japonicus* totivirus 1 (AejapTV1), *Ae. japonicus* anphevirus 1 (AejapAV1), and *Ae. japonicus* bunyavirus 1 (AejapBV1), respectively (Fig. 3). Based on comparisons to the closest reference, we observed that our assembly likely represented the full genome of AejapTV1 but that the genomes of AejapBV1 and AejapAV1 were not complete. In Supplementary Fig. S1, the putative genome organizations of AejapBV1 and AejapAV1 based on the reference sequences are shown. All three new viruses were closely related to known ISVs (Fig. 3; Table 2), but their final classification requires further biological characterization. All four identified viruses, one known and three new, have RNA genomes, either single-stranded (of positive or negative polarity) or double-stranded (Table 2). We could not identify sequences coding for polymerases among contigs belonging to the other two putative viruses, which we named *Ae. japonicus* bunyavirus 2 (AejapBV2) and *Ae. japonicus* rhabdovirus 1 (AejapRV1) based on the closest reference (Table 2, Supplementary Fig. S1B, S1D).

**Figure 4.**
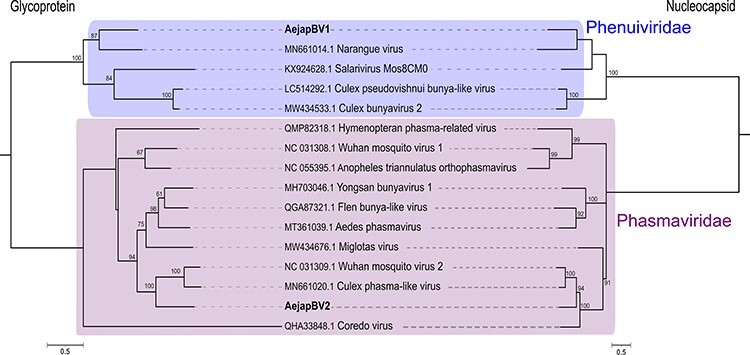
Phylogeny of glycoprotein and nucleocapsid of AejapBV1 and AejapBV2. Phylogenetic trees were generated using the glycoprotein and nucleocapsid amino acid sequences. The trees were inferred by using the maximum likelihood method. The trees with the highest log likelihood are shown. Number of conserved sites and the substitution models used for each tree: glycoprotein, 796 sites, WAG + G + F, and nucleocapsid, 573 sites, LG + G. Node bootstraps were calculated with 1,000 replicates and are shown close to each clade, and values < 60 were omitted. Trees were midpoint-rooted, and sequences from distinct viral families were included in the alignments. The trees are drawn to scale, and branch lengths represent expected numbers of substitutions per amino acid site. Accession numbers for the nucleotide sequences from which the corresponding protein sequences were derived or the direct protein sequences are shown with the virus names. Viruses identified in this study are in bold.

AejapBV2 contigs clustered with AejapTV1 based on co-occurrence in the four libraries (Fig. 2), but there is no further evidence that associates these sequences with the same virus. Instead, our overall analysis including small RNA size profiles (Fig. 2) and comparison of sequence similarity indicate that AejapBV2 and AejapTV1 are distinct viruses. Although no contigs corresponding to a putative segment L belonging to AejapBV2 were found in our libraries, we successfully assembled entire M and S genomic segments (Supplementary Figure S1B). The phylogenetic analysis of amino acid sequences shows that AejapBV2 segments coding for the glycoprotein and nucleocapsid are distant from AejapBV1-equivalent sequences (Fig. 4). Indeed, sequence similarity and phylogenetic analysis of nucleocapsid and glycoprotein of AejapBV2 indicate that this virus belongs to the family *Phasmaviridae* in contrast to AejapBV1 that belongs to the *Phenuiviridae* family (Fig. 4; Table 2). In an attempt to find sequences encoding an RdRp belonging to AejapBV2 in our libraries, we mapped reads from each library to the segment L sequence of the virus whose segments M and S were most similar to AejapBV2 (Table 2), Wuhan mosquito virus 2 (GenBank: NC_031312.1) (Supplementary Fig. S2). Even allowing multiple mismatches, we did not observe an expressive amount of reads nor a continuous coverage of the segment L of this virus.

**Figure 5.**
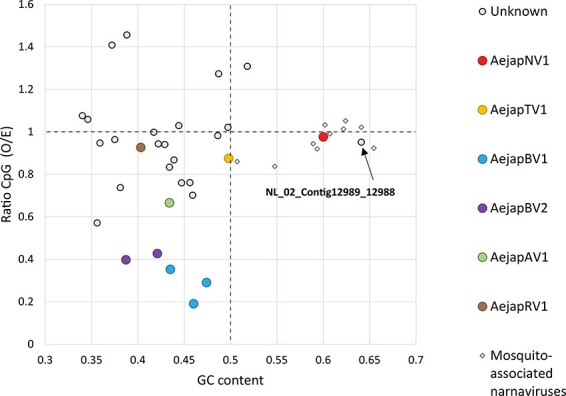
CpG dinucleotide usage and GC content of viral and unknown sequences discovered in *Ae. japonicus*. Data points indicate the ratio of GC content (*X*-axis) and CpG dinucleotide frequency (*Y*-axis) of individual viral or unknown sequences. The GC content of 0.5 (vertical dotted line) is the expected frequency if the genome would consist of 50 per cent GC and 50 per cent AT. The observed/expected (O/E) CpG ratio of 1.0 (horizontal dotted line) is the expected frequency of CpG occurrence when all mononucleotides in a given RNA sequence would be randomly distributed. Besides the viral and unknown sequences obtained from *Ae. japonicus*, the genome sequences of previously discovered narnaviruses in other mosquito species were also included in the analysis. GenBank accession numbers of these mosquito-associated narnaviruses are MW226855.1, MW226856.1, NC_035120.1, KF298284.2, MF176344.1, KF298275.2, MW520409.1, MK285331.1, MK285333.1, and MK285336.1.

To further characterize the identified viruses (Table 2), the (di)nucleotide usage of their genomes was analysed, as in particular CpG dinucleotide usage and GC content markedly differ between the genomes of distinct virus families (Auewarakul 2005; Cheng et al. 2013; Di Giallonardo et al. 2017). The largest representative sequence of each virus or viral segment identified was analysed (Table 2). The twenty-two contigs of unknown origin larger than 500 nt were also included to evaluate possible associations with the viruses found in our datasets. The L, M, and S segments of AejapBV1 and the M and S segments of AejapBV2 showed strong CpG under-representation (< ratio 0.4) and a relatively low GC content (< 50 per cent) (Fig. 5), which is typical for bunyaviruses (Di Giallonardo et al. 2017). AejapTV1 (yellow) greatly differed from these bunyaviruses with an almost unbiased CpG usage (ratio close to 1) and a GC content of approximately 50 per cent (Fig. 5), which are expected values for totiviruses and other viruses with dsRNA genomes (Rima and McFerran 1997; Simón, Cristina, and Musto 2021). This adds yet another piece of evidence at the sequence level that, despite clustering together based on co-occurrence (Fig. 2), AejapTV1 and AejapBV2 are indeed different viruses. The positive-stranded AejapNV1 had a higher GC content compared to the negative-stranded AejapBV1, AejapBV2, AejapAV1 , and AejapRV1 (Fig. 5). This is in accordance with a previous study in which positive-sense RNA viruses were shown to have significantly higher GC contents as compared to negative-sense RNA viruses (Auewarakul 2005). Interestingly, one of the unknown contigs (NL_02_Contig12989_12988; indicated by an arrow in Fig. 5) showed a high GC content and an unbiased CpG frequency. This contig grouped together with narnaviruses previously discovered in other mosquito species (diamond symbols) and AejapNV1 (Fig. 5). As shown before, this contig clustered together with AejapNV1 based on co-occurrence and small RNA profile (Fig. 2; in bold). These observations reinforce the idea that this unknown contig belongs to AejapNV1.

### Small RNA profiles and genome organization of AejapNV1

**Figure 6.**
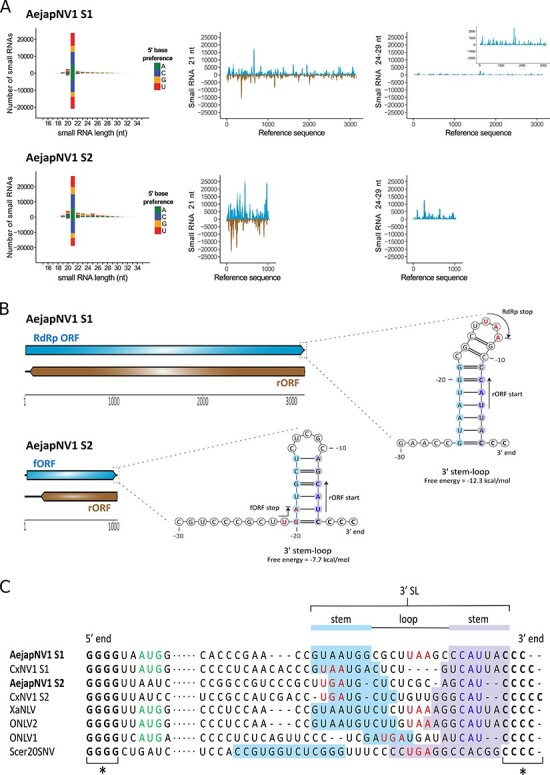
Small RNA profiles and genome organization of AejapNV1. (A) Left: size distribution and 5ʹ base preference of small RNAs derived from AejapNV1 S1 and S2. Middle and right: coverage of 21 and 24-29 nt sized small RNAs across S1 and S2. Viral reads mapping to the positive strand are shown in blue, whereas viral reads mapping to the negative strand are shown in brown. (B) Genome organisation of AejapNV1. The ambigrammatic coding strategy of S1 and S2 is shown. RdRp ORF and fORF on the positive strand are shown in blue, whereas reverse ORFs (rORFs) on the negative strand are shown in brown. Untranslated regions are indicated by black lines. Predicted SL structures at the 3ʹ terminus of the positive-sense RNA strand are also shown for both segments. The locations of start and stop codons are indicated by arrows and coloured blue and red, respectively. (C) Multiple sequence alignment of the 5ʹ and 3ʹ termini of the positive strand for indicated narnaviruses. Asterisks (*) indicate conserved, complementary runs of G or C nucleotides at the 5ʹ or 3ʹ end, respectively. Dots represent the remainder of the viral genome. Start codons of RdRp ORF are in green, stop codons of RdRp ORF/fORF are in red, and start codons of rORF are in blue. The start codons of the fORFs of AejapNV1 S2 and CxNV1 S2, as well as the start codon of the RdRp ORF of Scer20SNV, are located more than 10 nt downstream of the 5ʹ end and therefore not shown in the alignment. The nucleotides involved in 3ʹ SL formation are indicated.

The small RNA size profile, small RNA coverage, and ambigrammatic coding strategy of the ∼1-kb long unknown contig (NL_02_Contig12989_12988) were very similar to those of the ∼3-kb long AejapNV1 genome sequence coding for the RdRp (Fig. 6A, 6B). Together with the similarities noted before, these observations indicate that this contig is part of AejapNV1 and was thus named AejapNV1 segment 2 (S2), while the sequence encoding the RdRp is now referred to as segment 1 (S1) (Table 2). For S1, we observed a strong bias of mapped 24-29 nt small RNAs for the positive RdRp strand (Fig. 6A). Likewise, S2 also showed preference of mapped 24-29 nt small RNAs towards one specific strand (Fig. 6A). Analysis of the small RNA coverage profiles of AejapNV1 S1 and S2 for each small RNA length separately indicated a bias of small RNAs (18–35 nt in length) towards the same specific strand for each small RNA length, except for 21 nt small RNAs, which were found in similar quantities on both strands (Supplementary Fig. S3). This asymmetric pattern for small RNAs sized 18–20 nt and 22–35 nt is likely caused by non-specific degradation of viral RNAs, indicating the relative abundance and exposure of each strand (Han et al. 2011). For AejapNV1 S1, the small RNA bias was indeed towards the strand encoding the RdRp, which is considered the positive strand (Supplementary Fig. S3). Therefore, we propose that the positive strand of AejapNV1 S2 is the one to which most small RNAs mapped.

To further investigate whether putative S2 of AejapNV1 was indeed related to S1, the 5ʹ and 3ʹ termini of both segments were analysed for the presence of conserved narnaviral motifs. The *Alphanarnavirus* clade contains the prototypical narnaviruses such as *Saccharomyces cerevisiae* 20S RNA narnavirus (Scer20SNV) and the mosquito-associated narnaviruses containing an rORF. These viruses contain short, complementary runs of G and C nucleotides at their 5ʹ and 3ʹ termini, respectively, and have a conserved RNA stem-loop (SL) structure at their 3ʹ end (Fujimura and Esteban 2007; Dinan et al. 2020). In addition, it has very recently been described the mosquito-associated, ambigrammatic *Culex* narnavirus 1 (CxNV1) (Goertz et al. 2019) also consists of two segments (an RdRp segment, hereafter named S1, and a ‘Robin’ segment, hereafter named S2), both of which show these typical conserved features at the 5ʹ and 3ʹ genomic termini (Retallack et al. 2021).

Using a multiple sequence alignment, the terminal sequences of AejapNV1 S1 and S2 were compared with genomes from four ambigrammatic narnaviruses found in mosquitoes (CxNV1 S1, GenBank MW226855.1; CxNV1 S2, GenBank MW226856.1; Xanthi narna–like virus (XaNLV), GenBank MW520409.1; *Ochlerotatus*-associated narna-like virus 2 (ONLV2), GenBank KF298284.2; *Ochlerotatus*-associated narna-like virus 1 (ONLV1), GenBank KF298275.1) and the yeast-infecting Scer20SNV (GenBank NC_004051.1). For all viruses and segments analysed, short runs of G and C nucleotides were present at the 5ʹ and 3ʹ termini (Fig. 6C, indicated by asterisks). Based on RNA structure modelling, a conserved SL structure was predicted to occur at the 3ʹ terminus of AejapNV1 S1 and S2 (Fig. 6B, 6C). Similar conserved SL structures at the 3ʹ terminus, differing in size and with covarying base pairs in the stem region (Fig. 6C), have previously been observed for CxNV1 S1 and S2 (Retallack et al. 2021), ONLV1 and ONLV2 (Dinan et al. 2020), and Scer20SNV (Fujimura and Esteban 2007) and were also found for XaNLV (Supplementary Fig. S4). The presence of these conserved narnaviral characteristics in the genomic termini of both S1 and S2 of AejapNV1 not only indicated that our small RNA sequencing method was able to recover (near) complete genomic sequences but also corroborated the association between AejapNV1 S1 and the newly discovered S2.

### Insights into AejapNV1 S2 ORFs

To the best of our knowledge, the sequence of CxNV1 S2 is the only publicly available second segment sequence of a narnavirus infecting mosquitoes. Despite the similar genomic termini and ambigrammatic coding strategy (Fig. 6) (Retallack et al. 2021), the S2 segments of AejapNV1 and CxNV1 are very divergent, presenting ∼46 per cent of global identity at the nucleotide level. We therefore compared the putative polypeptides encoded by the S2 segment of these two viruses. Both forward ORFs (fORFs) and rORFs presented similar biochemical properties with high isoelectric points, suggesting a basic nature of these proteins (Supplementary Table 6). At the amino acid sequence level, we observed 29.34 per cent identity for fORFs and 26.89 per cent for rORFs (Supplementary Fig. S5). The predictions of secondary structures showed structured and disordered regions for the fORF-encoded proteins of both viruses, with the presence of many predicted α-helices in potentially homologous regions (Supplementary Fig. S5A). The same pattern was not observed for the rORFs (Supplementary Fig. S5B). Possible transmembrane regions were detected for both fORFs within AejapNV1 S2 residues 149 to 164 and CxNV1 S2 residues 99 to 114 (Supplementary Fig. S5A). Using AlphaFold, we could not obtain highly confident tertiary structure predictions (per-residue local distance difference test score (pLDDT) values > 90) for the entire proteins generated from the fORFs and rORFs of both viruses (Supplementary Fig. S6). At pLDDT values > 70, AlphaFold predicted a core structured region composed of α-helices shared by the fORFs of both viruses (Supplementary Fig. S6A, S6C), supporting the predicted organization of secondary structures shown in Supplementary Figure S5A.

### Small RNA profiles and genome organisation of AejapTV1

**Figure 7.**
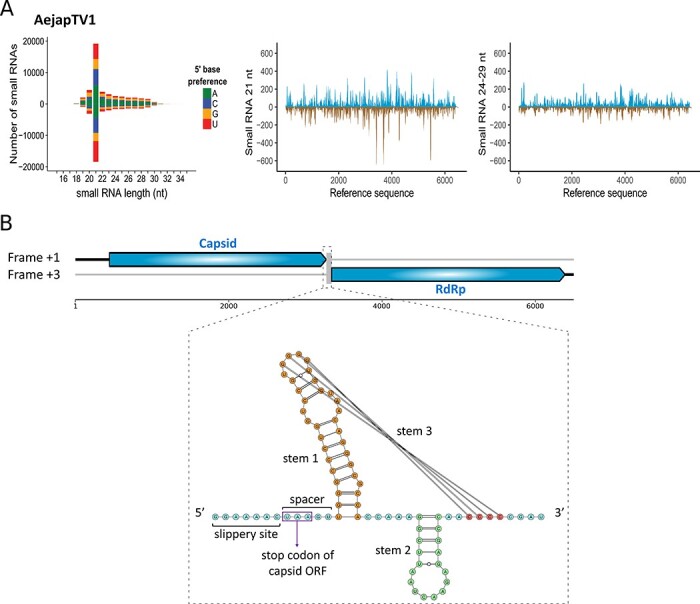
Small RNA profiles and genome organisation of AejapTV1. (A) Left: size distribution and 5ʹ base preference of small RNAs derived from AejapTV1. Middle and right: coverage of 21 and 24-29 nt sized small RNAs across the genome of AejapTV1. Viral reads mapping to the positive strand are shown in blue, whereas viral reads mapping to the negative strand are shown in brown. (B) Genome organization of AejapTV1. Untranslated regions are indicated by black lines. Capsid and RdRp ORFs on the positive strand are shown in blue. These ORFs are encoded in different frames, and a putative −1 ribosomal frameshift area was observed in between the two ORFs. This area consisted of a slippery heptamer, a spacer region, and a predicted three-stemmed pseudoknot with a free energy of −33.97 kcal/mol.

Small RNAs derived from AejapTV1 (Fig. 2; in yellow) showed a sharp 21 nt peak matching both strands (Fig. 7A), which suggests activation of the siRNA pathway and active RNA replication in mosquitoes. Small RNAs mapped along the entire assembled sequence (Fig. 7A), which contained a capsid ORF followed by an RdRp ORF (Fig. 7B). Interestingly, these two ORFs were encoded in different frames (Fig. 7B). Ribosomal frameshifting is a strategy commonly employed by members of the family *Totiviridae* during translation of their RdRp (Ghabrial and Nibert 2009; Firth and Brierley 2012). Therefore, it was investigated whether this strategy could potentially be employed by AejapTV1. Based on RNA structure modelling, a putative −1 ribosomal frameshift area was discovered at the end of the capsid ORF (Fig. 7B). A slippery site (GGAAAAC), present just before the stop codon of the capsid ORF, corresponded to the heptameric consensus motif typical for −1 ribosomal frameshifts and represents the area where the ribosome shifts back into another reading frame (Bekaert et al. 2003). Right after the slippery site, a spacer region of five nucleotides in length was found (Fig. 7B). This region was followed by a highly structured area consisting of a three-stemmed pseudoknot (Fig. 7B), which is expected to be responsible for pausing and relocating the ribosome. Similarly, a −1 ribosomal frameshift has also been predicted at the end of the capsid ORF for the mosquito-associated totiviruses *Armigeres subalbatus* totivirus and Omono River virus (Zhai et al. 2010; Isawa et al. 2011; Zhang et al. 2021), suggesting that −1 ribosomal frameshifting is a common feature of these mosquito-associated totiviruses.

### Small RNA profiles of AejapBV1, AejapBV2, AejapAV1, and AejapRV1

**Figure 8.**
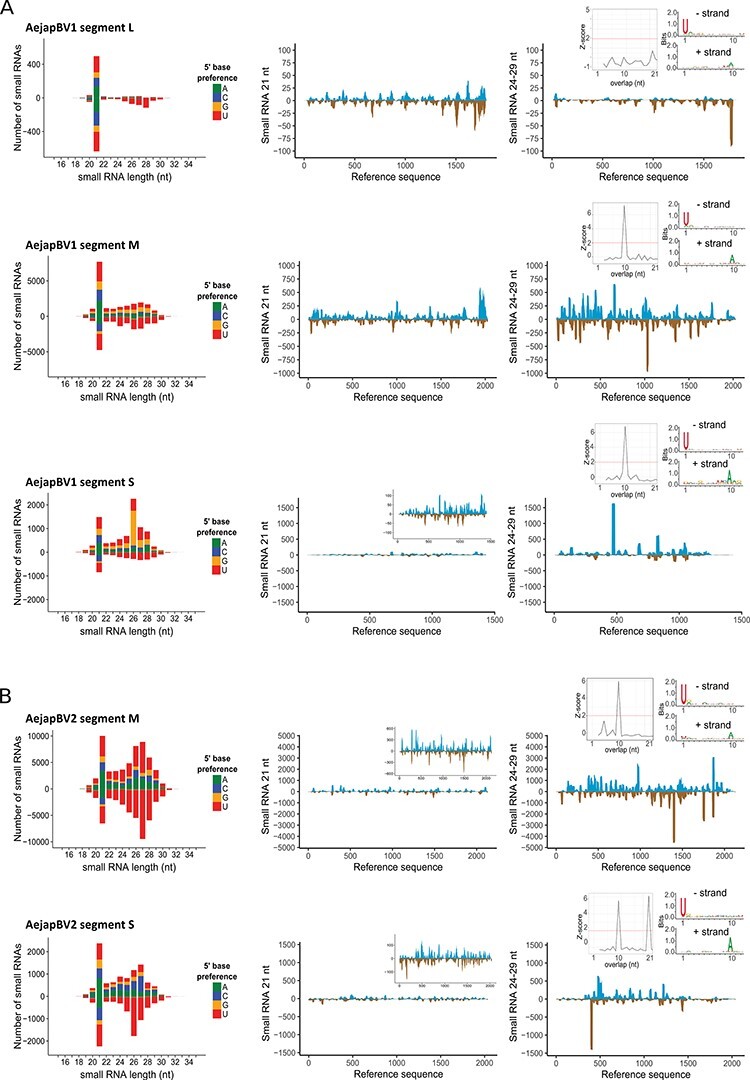
Small RNA profiles of AejapBV1 and AejapBV2. Size distribution and 5ʹ base preference of small RNAs derived from (A) AejapBV1 segments L, M and S and (B) AejapBV2 segments M and S are shown on the left, whereas coverage of 21 and 24-29 nt sized small RNAs across the same segments of the respective viruses is shown in the middle and on the right. For the coverage profiles, viral reads mapping to the positive strand are indicated in blue, whereas viral reads mapping to the negative strand are indicated in brown. Inset figures in the middle figures show a zoomed in 21 nt small RNA coverage. Inset figures in the right figures show features of 24-29 nt reads aligned to its respective viral references. Left inset figures show a histogram with normalized frequency of overlap sizes of reads aligned to positive and negative strands. Right inset figures show sequence logo representations of reads aligned to positive and negative strand separately.

**Figure 9.**
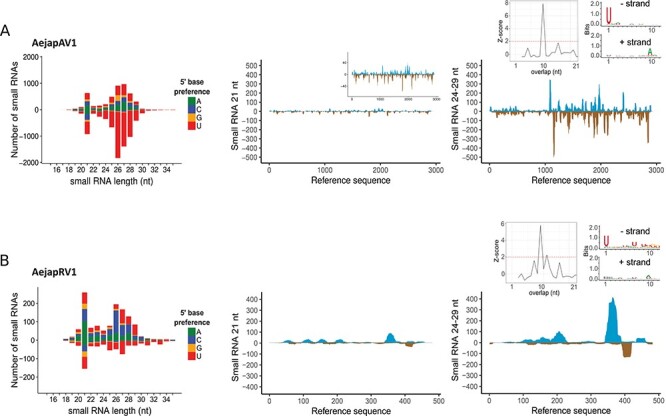
Small RNA profiles of AejapAV1 and AejapRV1. Size distribution and 5ʹ base preference of small RNAs derived from (A) AejapAV1 and (B) AejapRV1 are shown on the left. In the middle and on the right, the coverage of 21 and 24-29 nt sized small RNAs across the assembled contigs of the same respective viruses is shown. For the coverage profiles, viral reads mapping to the positive strand are indicated in blue, whereas viral reads mapping to the negative strand are indicated in brown. The inset figure in the middle figure of (A) shows a zoomed in 21 nt small RNA coverage. Inset figures in the right figures show features of 24-29 nt reads aligned to its respective viral references. Left inset figures show a histogram with normalized frequency of overlap sizes of reads aligned to positive and negative strands. Right inset figures show sequence logo representations of reads aligned to positive and negative strand separately.

For the two bunyaviruses in our datasets, AejapBV1 and AejapBV2, a peak consisting of 21 nt small RNAs (characteristic of siRNA pathway activation) was observed for all three (Fig. 8A) or two (Fig. 8B) segments, respectively, and siRNAs aligned across entire segments. Also, RNAs with characteristics of piRNAs (∼24-29 nt in length) were detected for both viruses (Fig. 8A, B). Those RNAs showed U enrichment at the first nt of antisense reads and A enrichment at the tenth nt of sense reads, further suggesting their piRNA biogenesis (Fig. 8A, B). Moreover, reads that mapped to the segments M and S from both viruses presented a significant 10-nt overlap between small RNAs from sense and antisense strands, which is in accordance with the piRNA signature of the ping-pong amplification mechanism (Miesen, Joosten, and van Rij 2016). In a similar fashion to AejapBV1 and AejapBV2, small RNAs derived from AejapAV1 and AejapRV1 also showed a 21 nt siRNA peak, as well as ∼24-29 nt sized piRNAs with ping-pong amplification features (Fig. 9A, B). In the case of AejapRV1, this pattern reinforces the idea that we identified a real exogenous rhabdovirus that is replicating in *Ae. japonicus* rather than an EVE in the mosquito genome, despite the absence of an RdRp sequence.

### Prevalence of viruses in field-collected *Ae. japonicus* mosquitoes

**Figure 10.**
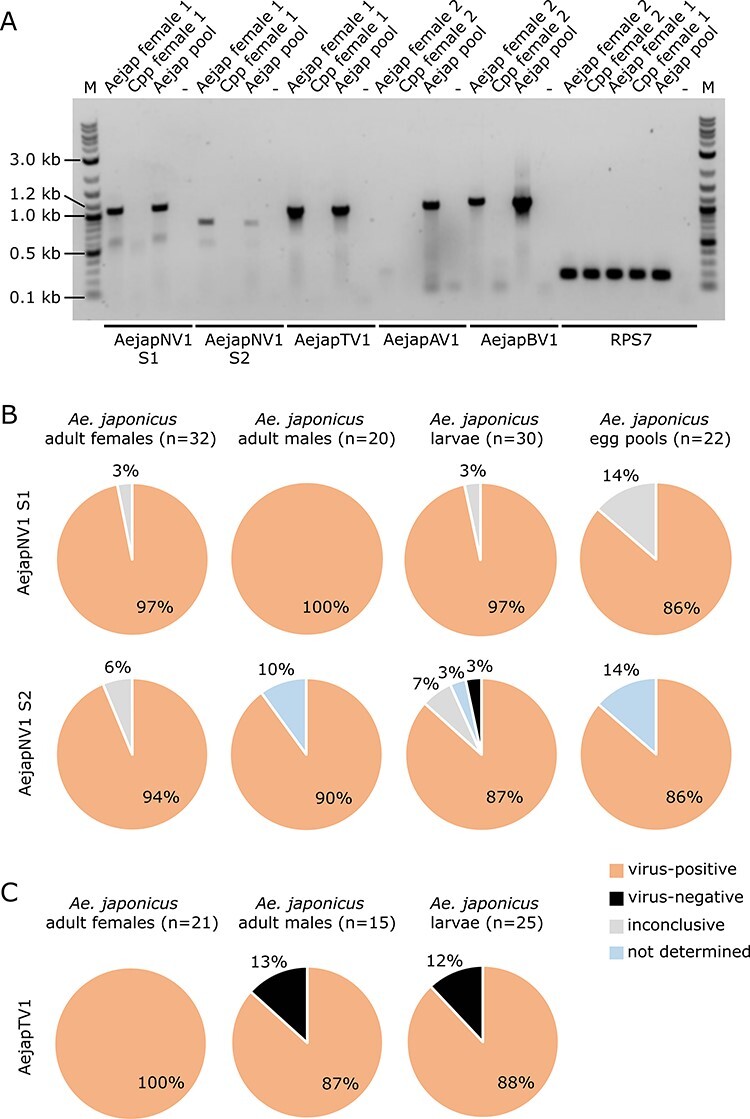
Detection of AejapNV1 S1 and S2, AejapTV1, AejapAV1, and AejapBV1 in field-collected *Ae. japonicus* from the Netherlands by RT-PCR. (A) Individual adult female of *Ae. japonicus* (Aejap individual females, 1 and 2) or *Cx. pipiens* (Cpp individual females, 1 and 2), a pool of four *Ae. japonicus* adult females (indicated by ‘Aejap pool’), and a water sample (no RNA; negative control, indicated by ‘-’) were tested for the presence of the RdRp segment of the four viruses we identified. All samples were also tested for mosquito RPS7 to check the quality of the RNA. Expected amplicon sizes were 1060 bp (AejapNV1 S1), 774 bp (AejapNV1 S2), 998 bp (AejapTV1), 1043 bp (AejapAV1), 1095 bp (AejapBV1), and 175 bp (RPS7). Aejap individual Female 1 tested positive for AejapNV1 S1 and S2 and also for AejapTV1. Aejap individual Female 2 tested negative for AejapAV1, but positive for AejapBV1. The pool of *Ae. japonicus* females was positive for all tested viruses. *Culex pipiens* females tested negative for all viruses. The lanes indicated with ‘M’ contain the DNA marker. (B) Prevalence of AejapNV1 S1 and S2 in *Ae. japonicus* adult females, adult males, larvae, and pools of twenty-five eggs. The number of samples tested is indicated by ‘*n*’. Individual samples were tested twice for each virus by RT-PCR. Samples with different results in the first and second tests were considered ‘inconclusive’. ‘Not determined’ refers to samples teste for S1 that we did not have enough RNA for additional testing for the presence of S2. (C) Prevalence of AejapTV1 in *Ae. japonicus* adult females, adult males, and larvae. Individual samples were screened by RT-PCR. The number of samples tested is indicated by ‘*n*’.

To confirm whether the *de novo* assembled viruses were present in field-collected *Ae. japonicus*, RT-PCR assays were designed using primers targeting the RdRp coding region of AejapNV1, AejapTV1, AejapAV1, or AejapBV1. RT-PCRs with primers targeting S2 of AejapNV1 were also performed to confirm the presence of this putative segment. All four viruses were successfully detected in individual samples and pools of field-collected adult *Ae. japonicus* females, while they could not be detected in adult *Cx. pipiens* females that were tested in parallel (Fig. 10A). For AejapNV1, the presence of both S1 and S2 in *Ae. japonicus* was confirmed (Fig. 10A). DNA sequencing of the AejapNV1 S2 RT-PCR product showed 99.6 per cent identity to the contig of AejapNV1 S2 previously obtained from *de novo* small RNA assembly. These analyses confirm that our metagenomic approach successfully identified viruses and did not generate artefacts of assembly.

Next, to further evaluate the virus circulation in natural conditions, we screened approximately 100 individual samples of field-collected *Ae. japonicus* including adult females, adult males, larvae, and egg pools for the presence of AejapNV1 and AejapTV1 (except for egg pools). Both segments of AejapNV1 were present at very high prevalence (close to 100 per cent) in all tested mosquito samples and the different life stages (Fig. 10B). AejapTV1 was also found at high prevalence with 100 per cent virus-positive adult females, 87 per cent virus-positive adult males, and 88 per cent virus-positive larvae (Fig. 10C).

## Discussion

In this study, we analysed the virome of field-collected *Ae. japonicus* and found four viruses, three of them novel species, and another two putative novel viruses associated with this invasive vector mosquito. We applied a small RNA-based metagenomic approach previously developed by our group (Aguiar et al. 2015) that allowed us to overcome three main challenges of eukaryotic viral metagenomics: (1) the differentiation of replicating exogenous viruses from EVEs based on the presence of siRNAs, (2) the association of different segments of the same virus based on co-occurrence and small RNA profiles, and (3) the identification and classification of highly divergent viral sequences that do not have similarity to any known reference sequence.

All viruses detected in *Ae. japonicus* have RNA genomes (Abbo et al. 2020; Faizah et al. 2020). Detection of DNA viruses using RNA-seq approaches is not supposed to be a limitation since DNA viruses produce different RNA molecules during their replication cycle (de Faria et al. 2022). Indeed, DNA viruses have been successfully identified in mosquitoes via metatranscriptomics approaches based on long and small RNA sequencing (Ma et al. 2011; Feng et al. 2022). From a broader perspective, DNA viruses seem to be under-represented in mosquito viromes and in eukaryotes as a whole (Krupovic, Dolja and Koonin 2023; de Almeida et al. 2021). Therefore, DNA viruses may indeed be absent in the *Ae. japonicus* virome rather than undetected due to methodological limitations.

Regarding our results on the identification of *Ae. japonicus*–associated viruses, AejapNV1 is a good example of the power of our strategy. *De novo* assembly of small RNAs did not only yield the ∼3-kb long primary genome segment for AejapNV1 but interestingly also revealed the presence of a ∼1-kb long secondary genome segment (also ambigrammatic, but containing ORFs with no hits in the databases), which we could confidently associate with the same virus. Small RNA abundancy and size distribution profiles were very similar for both segments, suggesting that they belong to the same virus. Further support comes from the similar CpG and GC content, conserved genomic termini, and unusual ambigrammatic nature of segments S1 and S2. Bisegmented narnaviruses have also been discovered in *Plasmodium* (Charon et al. 2019), in a trypanosomatid (Lye et al. 2016; Grybchuk et al. 2018), and, very recently, in mosquitoes (Batson et al. 2021; Dudas et al. 2021). For the mosquito-associated, bisegmented, ambigrammatic CxNV1, the presence of the primary RdRp segment was found to be required for replication of the secondary (‘Robin’) segment in *Culex* mosquito cells, whereas the primary segment could persist in cell culture without the presence of the secondary segment (Retallack et al. 2021). In our study, the primary and secondary genome segment of AejapNV1 co-occurred at very high frequency in field-collected *Ae. japonicus* mosquitoes, which is in accordance with previous findings for CxNV1 in wild-caught mosquitoes (Batson et al. 2021), thus suggesting that the secondary segment is of key importance for virus maintenance in the field.

Despite some conserved features between AejapNV1 S2 and CxNV1 S2, such as the genomic termini and ambigrammatic ORF structure, these sequences are highly divergent at nucleotide and amino acid sequence levels. Such divergence raises questions about the origin and function of this secondary genome segment. Here we analysed the conservation between polypeptides encoded by AejapNV1 S2 and CxNV1 S2 at the structure levels. Conserved motifs at secondary and tertiary levels between proteins encoded by the fORFs were observed when comparing the presence and synteny of predicted α-helix regions and the core structure predicted by AlphaFold. Interestingly, the rORF of CxNV1 S2 may not encode a functional protein (Dudas et al. 2021). Nevertheless, the function of the secondary genome segment of AejapNV1 and CxNV1 remains unknown, and future studies are needed to elucidate its role and evolutionary origin. These narnavirus secondary segments belong to the dark matter of metagenomics, since sequence similarity methods, such as local alignments, were not able to associate the AejapNV1 S2 sequence with the CxNV1 S2 reference in GenBank. Even the application of the powerful tool AlphaFold (Jumper et al. 2021) to predict tertiary structures and infer protein function was limited by the lack of similar sequences to ORFs in AejapNV1 S2. AlphaFold relies on initial alignments to protein sequence and structure databases, such as Uniref90 and the Protein Data Bank, to acquire atomic tridimensional data and perform structural assessments (Jumper et al. 2021). This could also explain the lack of confident predictions for our fORFs and also for any attempt of predicting structures for highly divergent sequences. This is a good example of how bioinformatics pipelines for viral sequence identification in metagenomic data still have room for improvement. Increased knowledge about the viral metagenomic dark matter is paramount to allow large-scale automated generalizations of the evolution and function of divergent viral sequences. Of note, our clustering approach based on small RNA profiles and co-occurrence was critical to identify the unknown contig corresponding to Segment 2 of AejapNV1. This reinforces the power of small RNA-based approaches to recover viral sequences from the metagenomic dark matter (Aguiar et al. 2015).

Still, the origin of a substantial portion of the dark matter present in *Ae. japonicus* remains enigmatic. Despite the presence of an siRNA profile, we could not clearly associate twenty-one unknown contigs to any defined viral species in this study. Further studies will be necessary to determine if these unknown sequences with the siRNA profile are of viral origin. One possible explanation derives from the lack of a reference genome available for *Ae. japonicus*, implying that some of those unknown contigs could have originated from unknown repetitive elements in the genome and/or overlapping mRNA fragments that may produce endogenous siRNAs (Ghildiyal et al. 2008).

Our small RNA-based approach also pointed to the presence of two distinct bunyaviruses in *Ae. japonicus*. However, we could not identify a potential L segment encoding an RdRp for AejapBV2. Based on previous successful detection of segmented viruses from different families including bunyaviruses using our small RNA-based approach (Aguiar et al. 2015; Olmo et al. 2023), it is unlikely that Segment L of AejapBV2 was present in the samples and remained undetected. In addition, we successfully assembled complete M and S segments for AejapBV2. Segment reassortments between bunyaviruses are frequently described, and it has been suggested that most if not all bunyaviruses currently present have arisen from reassortments (Briese, Calisher, and Higgs 2013; Kapuscinski et al. 2021). Given the ability of bunyaviruses to reassort genome segments, AejapBV2 could possibly use the RdRp of AejapBV1 for replication. However, AejapBV1 and AejapBV2 belong to two different bunyavirus families, *Phenuiviridae* and *Phasmaviridae*, respectively, which might render complementation or reassortment less likely. In addition, this hypothesis implies that AejapBV2 replication is dependent on the presence of the L segment of AejapBV1, but we could not detect AejapBV1 in our *Ae. japonicus* NL_02 small RNA library, while AejapBV2 was present (Fig. 2). Future studies will be needed to elucidate the replication strategy of AejapBV2 in *Ae. japonicus*.

Remarkably, AejapBV1, AejapBV2, AejapAV1, and AejapRV1 produced abundant piRNAs with strong signals of a ping-pong mechanism. Notably, not all viruses induce the production of piRNAs during infection in mosquitoes (Miesen, Joosten, and van Rij 2016; Varjak, Leggewie, and Schnettler 2018). A similar small RNA profile was described for another bunyavirus, *Phasi Charoen–like virus*, infecting *A. aegypti*, and the authors reasoned that the piRNA biogenesis could be linked to ovary tropism (Aguiar et al. 2015). It would be of interest to investigate if the piRNA profiles observed in our study correlate with tissue tropism in *Ae. japonicus*. Interestingly, there is a striking difference in the profile of piRNAs produced from Segments M and S of AejapBV1 and AejapBV2, which suggests that these viruses infect different tissues in the mosquito (Aguiar et al. 2015).

Screening of wild-caught *Ae. japonicus* from the Netherlands indicated a high prevalence of AejapNV1 and AejapTV1 across all tested mosquito life stages, suggesting efficient transmission of these viruses, possibly vertically. Such a phenomenon was described for narnaviruses discovered in fungi (Espino-Vazquez et al. 2020) and nematodes (Richaud et al. 2019), for which vertical transmission has been shown. The high percentage of mosquitoes that tested positive for AejapNV1 in our study is also in accordance with a recent study in Japan, where all tested *Ae. japonicus* mosquitoes harboured AejapNV1 (Faizah et al. 2020).

In our study, AejapNV1, AejapTV1, AejapAV1, AejapBV1, and AejapBV2 were all detected in *Ae. japonicus* mosquitoes from the Netherlands and France. This indicates a high stability of the virome across mosquito populations, which has also been observed for other mosquito species (Zakrzewski et al. 2018; Shi et al. 2020), and may suggest that these viruses have co-evolved with their mosquito host. Although the newly discovered virus species belong to different virus families and are thus highly diverse, they form a stable viral community in *Ae. japonicus* despite active antiviral RNAi responses, hence suggesting that these viruses should be considered important constituents of the biology of *Ae. japonicus* mosquito populations.

## Conclusions

This study uncovered the virome of the invasive Asian bush mosquito *Ae. japonicus*, a species that can transmit multiple arboviruses and has become increasingly prevalent in North America and Europe. We discovered a highly diverse virome in *Ae. japonicus* with viruses from at least five families: *Narnaviridae, Totiviridae, Xinmoviridae, Phenuiviridae*, and *Phasmaviridae*. We showed a collection of viruses not only diverse at the sequence level but also in RNAi responses generated by *Ae. japonicus*, providing the basis for future studies to elucidate the immune mechanisms involved in dealing with multiple viral infections. By discovering and characterizing AejapNV1 S2, we showed the strength of our small RNA-based approach to access the ‘viral dark matter’ of insect metagenomes. Beyond associating a highly divergent viral genomic segment with a single virus, we provided evidence that it replicates in the host by showing its siRNA signature. Our study establishes solid ground to explore whether these resident viruses could impact arbovirus transmission by *Ae. japonicus*. Infection with these likely ISVs might add yet another complex variable to the risk assessment of arbovirus outbreaks caused by *Ae. japonicus*, and future studies are needed to dissect the role of the virome in arbovirus transmission.

## Supplementary Material

vead041_Supp

## Data Availability

The small RNA datasets generated and/or analysed during the current study are available in the NCBI SRA under BioProject PRJNA545039 and PRJNA785589. The genome sequences of newly discovered viruses can be found in GenBank under NCBI accession numbers OP737829–OP737837.
