# Supplementary material for: The virome of the invasive Asian bush mosquito Aedes japonicus in Europe
Source: Virus Evol. 2023 Jul 3;9(2):vead041. doi: 10.1093/ve/vead041 (PMC10460169; doi:10.1093/ve/vead041)
Supplement: vead041_Supp [file vead041_supp.zip › suppl_data/Supplementary_figures_withlegends.pdf]

Supplementary Figures

The virome of the invasive Asian bush mosquito *Aedes japonicus* in Europe

Sandra R. Abbo[1,*], João P. P. de Almeida[2,*], Roenick P. Olmo[3], Carlijn Balvers[1,4], Jet S. Griep[1,4], Charlotte Linthout[4], Constantianus J. M. Koenraadt[4], Bruno M. Silva[2], Jelke J. Fros[1], Eric R. G. R. Aguiar[2,5], Eric Marois[3], Gorben P. Pijlman[1,#], João T. Marques[2,3,#]

* Contributed equally.

[#] Corresponding author.

[1] Laboratory of Virology, Wageningen University & Research, Wageningen, the Netherlands.

[2] Department of Biochemistry and Immunology, Instituto de Ciências Biológicas, Universidade Federal de Minas Gerais, Belo Horizonte, Brazil.

[3] Université de Strasbourg, CNRS UPR9022, INSERM U1257, Strasbourg, France.

[4] Laboratory of Entomology, Wageningen University & Research, Wageningen, the Netherlands.

[5] Department of Biological Science, Center of Biotechnology and Genetics, State University of Santa Cruz, Ilhéus, Brazil.

Corresponding authors emails: gorben.pijlman@wur.nl (G.P.J) and jtm@ufmg.br (J.T.M.)

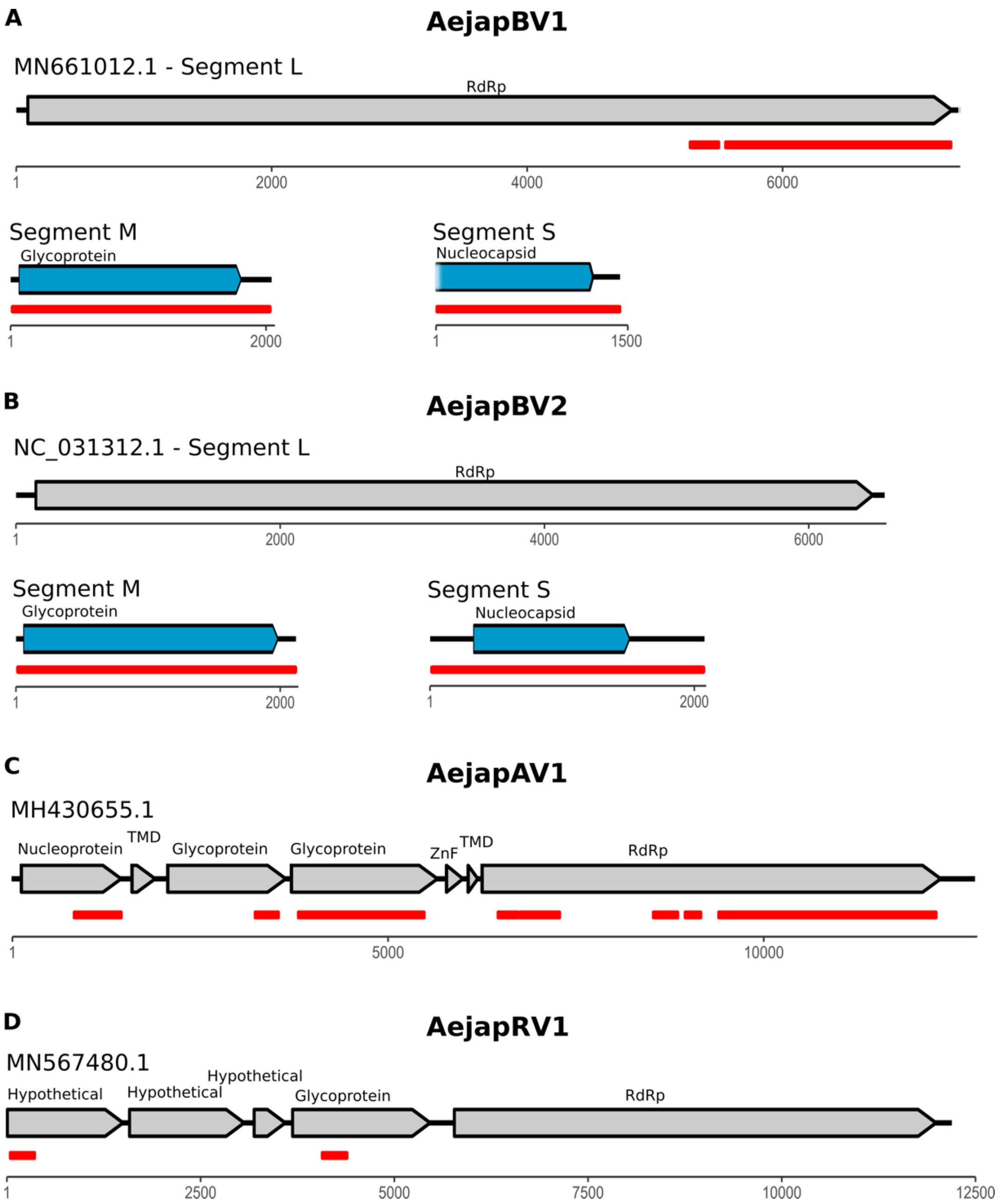

Supplementary Figure 1. Genomic organization of partially assembled viruses. Grey arrows represent ORFs from the closest GenBank reference viral sequence. Red lines indicate viral reference genome regions covered by our assembled contigs. Blue arrows represent ORFs from completely assembled viral segments in this work. Black lines indicate untranslated regions. **(A)** AejapBV1. The lack of an assembled 5' UTR for segment S is represented as a fading color region. Despite the lack of a 5' UTR and a start codon, the total ORF size of segment S is similar to its closest sequence in GenBank (QHA33859.1). **(B)** AejapBV2, **(C)** AejapAV1, **(D)** AejapRV1.

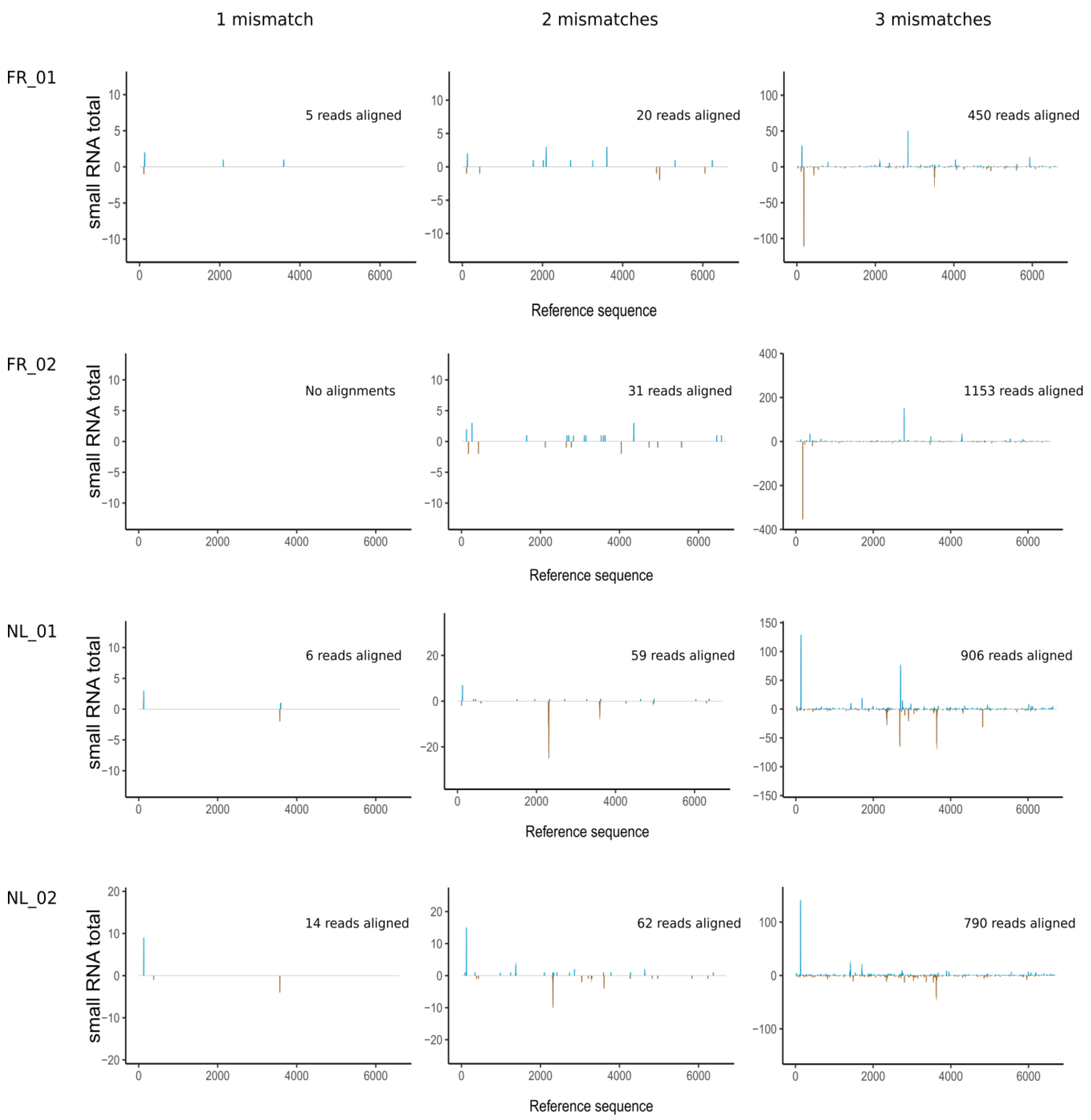

Supplementary Figure 2. Small RNA coverage of Wuhan mosquito virus 2 segment L. Total small RNA reads from each library (rows) were aligned to Wuhan mosquito virus 2 segment L (NC_031312.1), a potential homologous sequence of the missing segment L of our identified AejapBV2. In order to confirm the absence of a highly divergent segment L inexplicably not assembled, we aligned each small RNA library allowing one to three mismatches per read (columns) by varying the parameter *-v* of bowtie. Each panel represents the coverage and total reads assembled for the combination of one library and the maximum number of mismatches allowed per read. The blue lines indicate small RNA read coverage of forward strand and the brown lines of negative strand. Only when three mismatches were allowed, considerable numbers of reads aligned to the reference. Although with no signal of continuous coverages, these results likely indicate spurious alignments.

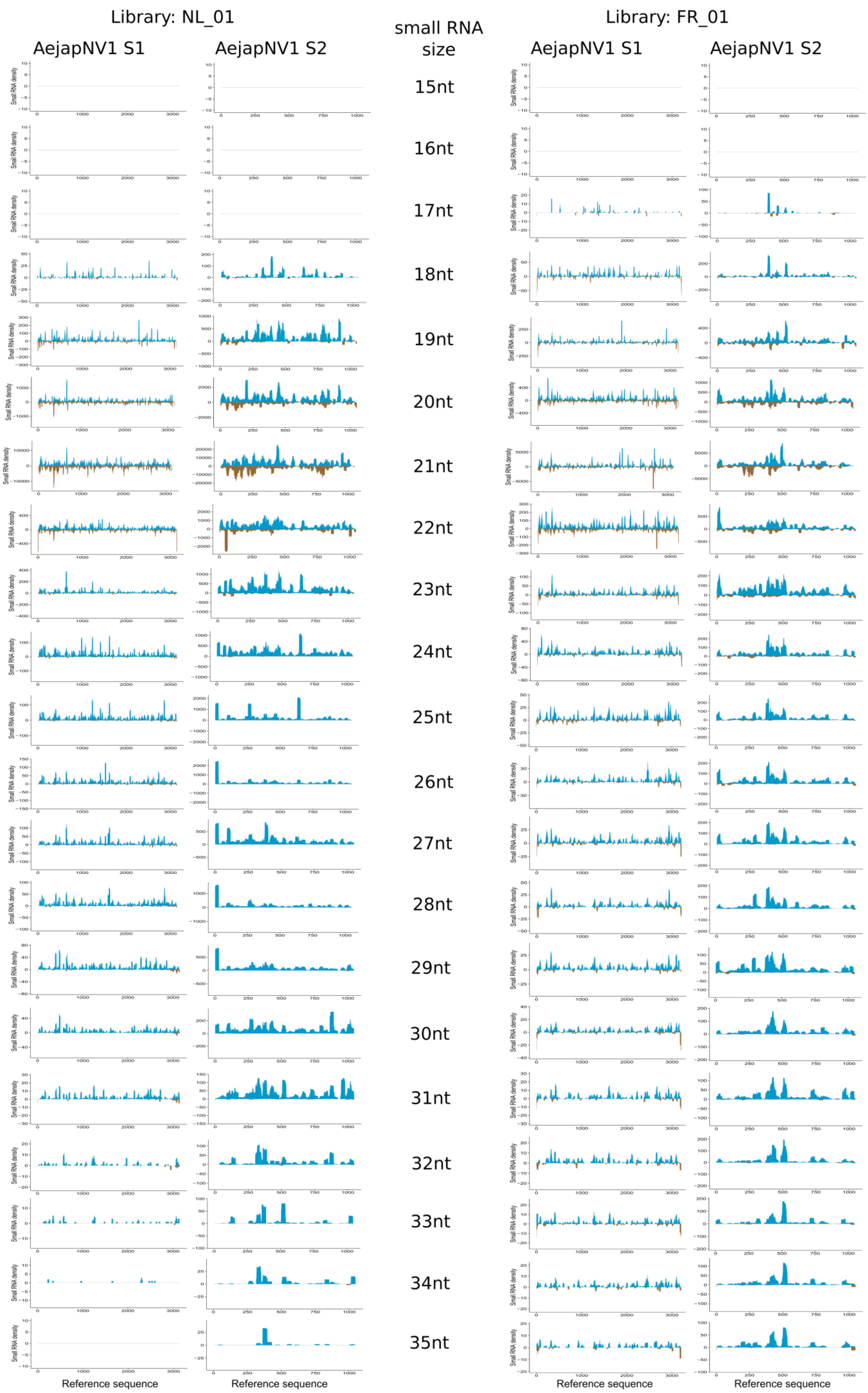
Library: NL_01
AejapNV1 S1
AejapNV1 S2
small RNA size
Library: FR_01
AejapNV1 S1
AejapNV1 S2
15nt
16nt
17nt
18nt
19nt
20nt
21nt
22nt
23nt
24nt
25nt
26nt
27nt
28nt
29nt
30nt
31nt
32nt
33nt
34nt
35nt
Small RNA density
Reference sequence
Reference sequence
Reference sequence
Reference sequence

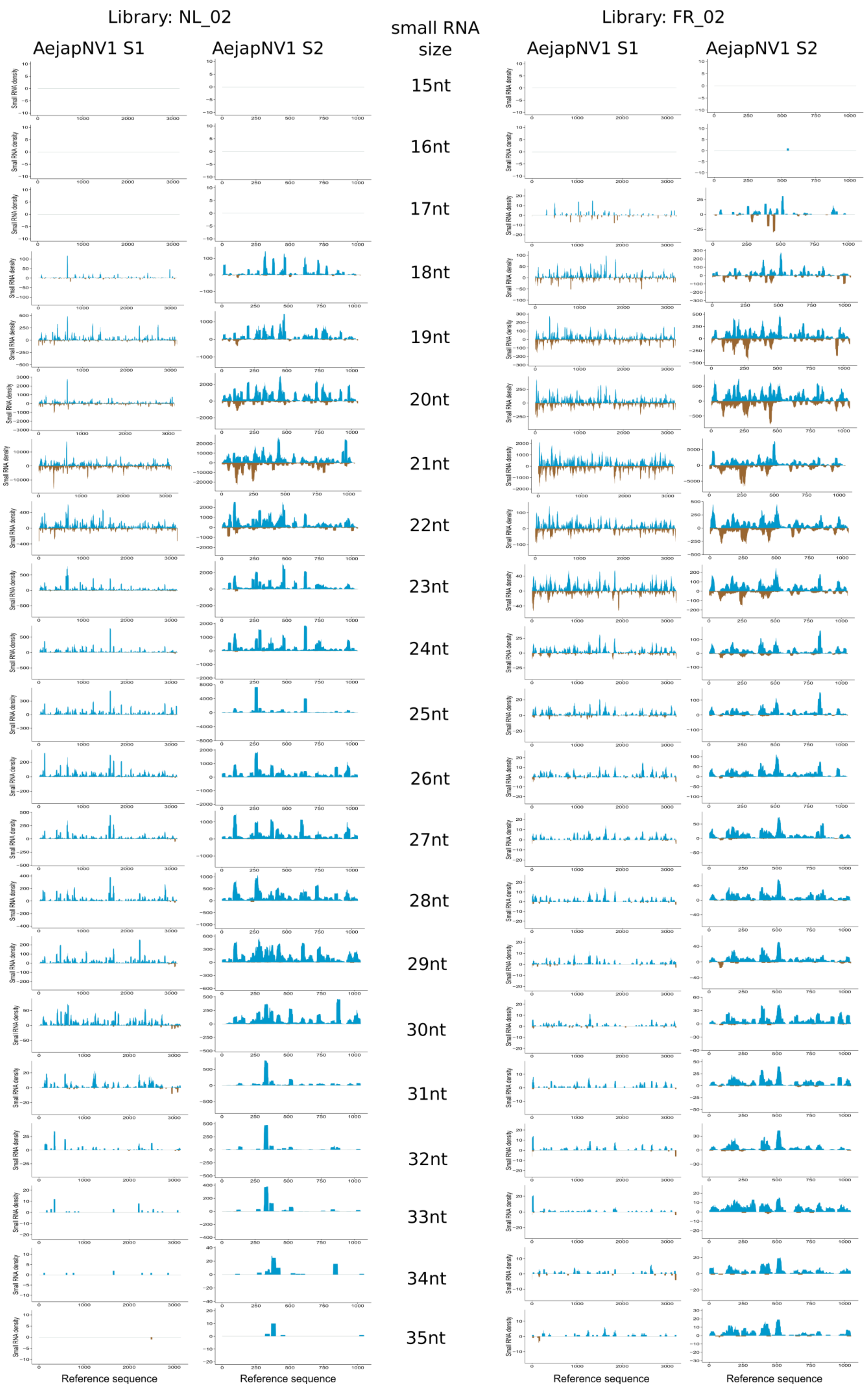
Library: NL_02
AejapNV1 S1
AejapNV1 S2
small RNA size
Library: FR_02
AejapNV1 S1
AejapNV1 S2
15nt
16nt
17nt
18nt
19nt
20nt
21nt
22nt
23nt
24nt
25nt
26nt
27nt
28nt
29nt
30nt
31nt
32nt
33nt
34nt
35nt
Small RNA density
Reference sequence
Reference sequence
Reference sequence
Reference sequence

Supplementary Figure 3. Strand-specific small RNA coverage bias of AejapNV1 S1 and S2. Reads from sizes 15 to 35 nt from each library were aligned separately to segments S1 and S2. The blue area indicates small RNA read coverage of forward strand and the brown area of negative strand. The S1 sequence orientation was determined based on the RdRp coding ORF direction. A small RNA coverage bias towards the positive RdRp strand of S1 was seen, and a similar coverage bias was seen for S2, thus indicating the putative positive strand.

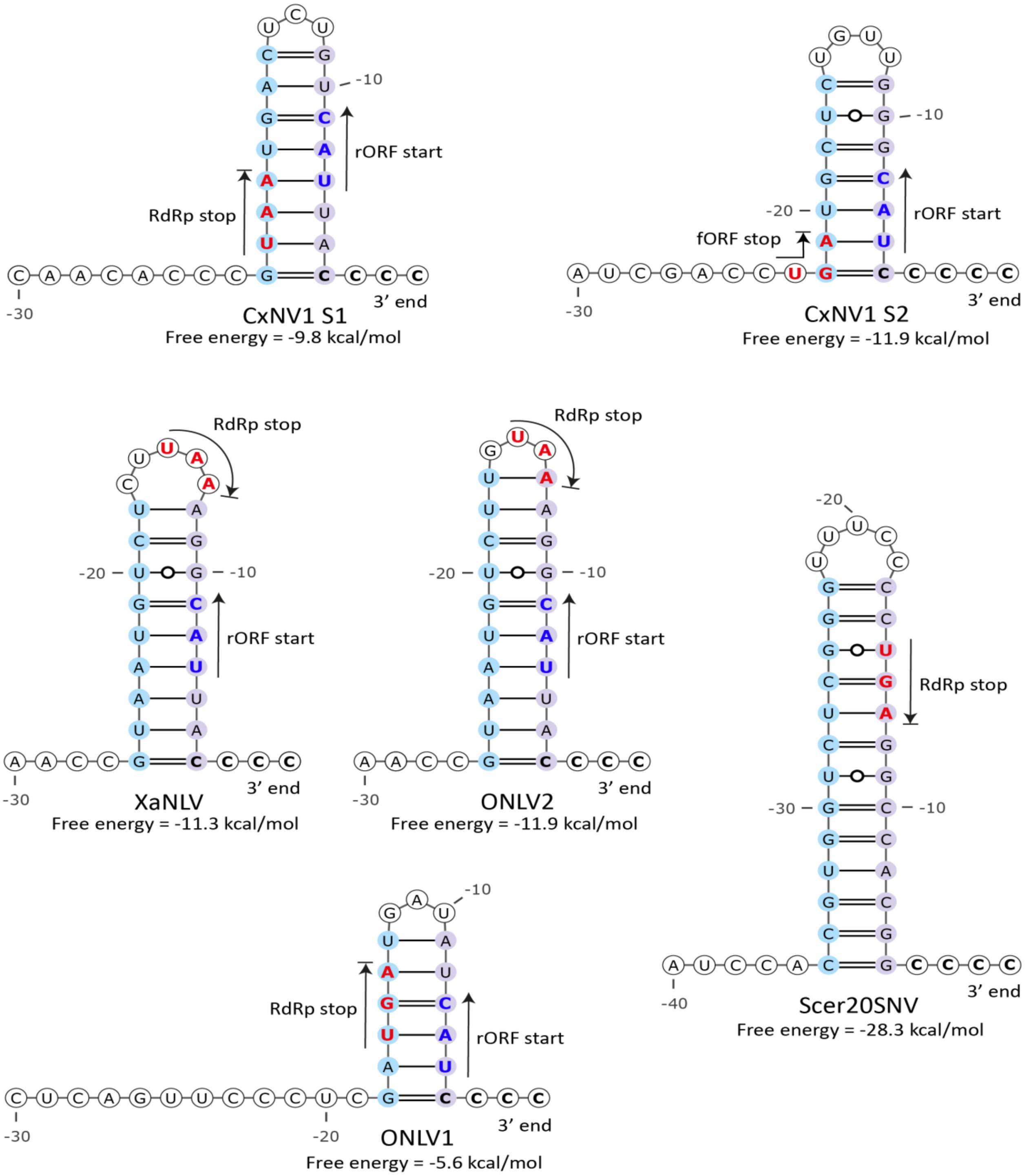

Supplementary Figure 4. Predicted 3' stem-loop structures of CxNV1 S1, CxNV1 S2, XaNLV, ONLV2, ONLV1 and Scer20SNV. The predicted RNA structures at the 3' terminus of the positive-sense RNA strand are shown. Locations of start and stop codons are indicated by arrows and colored blue and red, respectively.

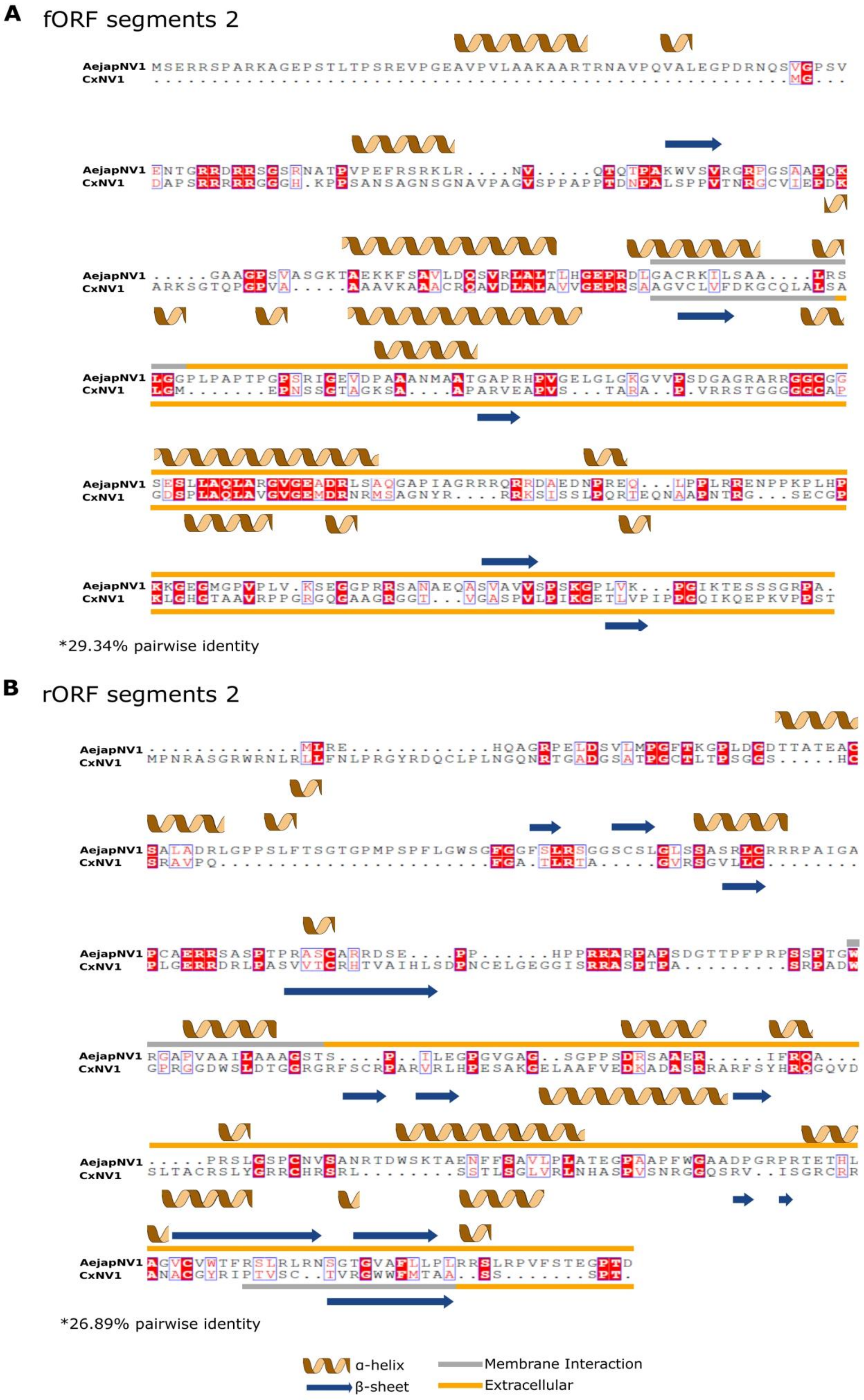

Supplementary Figure 5. Primary and secondary protein structure comparisons of segment

2 ORFs from AejapNV1 and CxNV1. Sequence alignment of AejapNV1 and CxNV1 (GenBank MW226856.1) fORF and rORF segment 2 and its respective secondary structure prediction and membrane interaction. In **(A)** are shown the comparisons of fORFs and in **(B)** the rORFs. Residues with identical matches are colored in red boxes, and residues with similar side chain physical-chemical properties are highlighted with a blue box and written in red. Above the sequences are represented the PSIPRED and MEMSAT-SVM secondary structure prediction results of AejapNV1, and under the sequences, the CxNV1 prediction. The α-helix regions are represented with brown helices and the β-strands with dark blue arrows. The regions with predicted membrane interaction are in gray boxes and predicted extracellular regions in yellow.

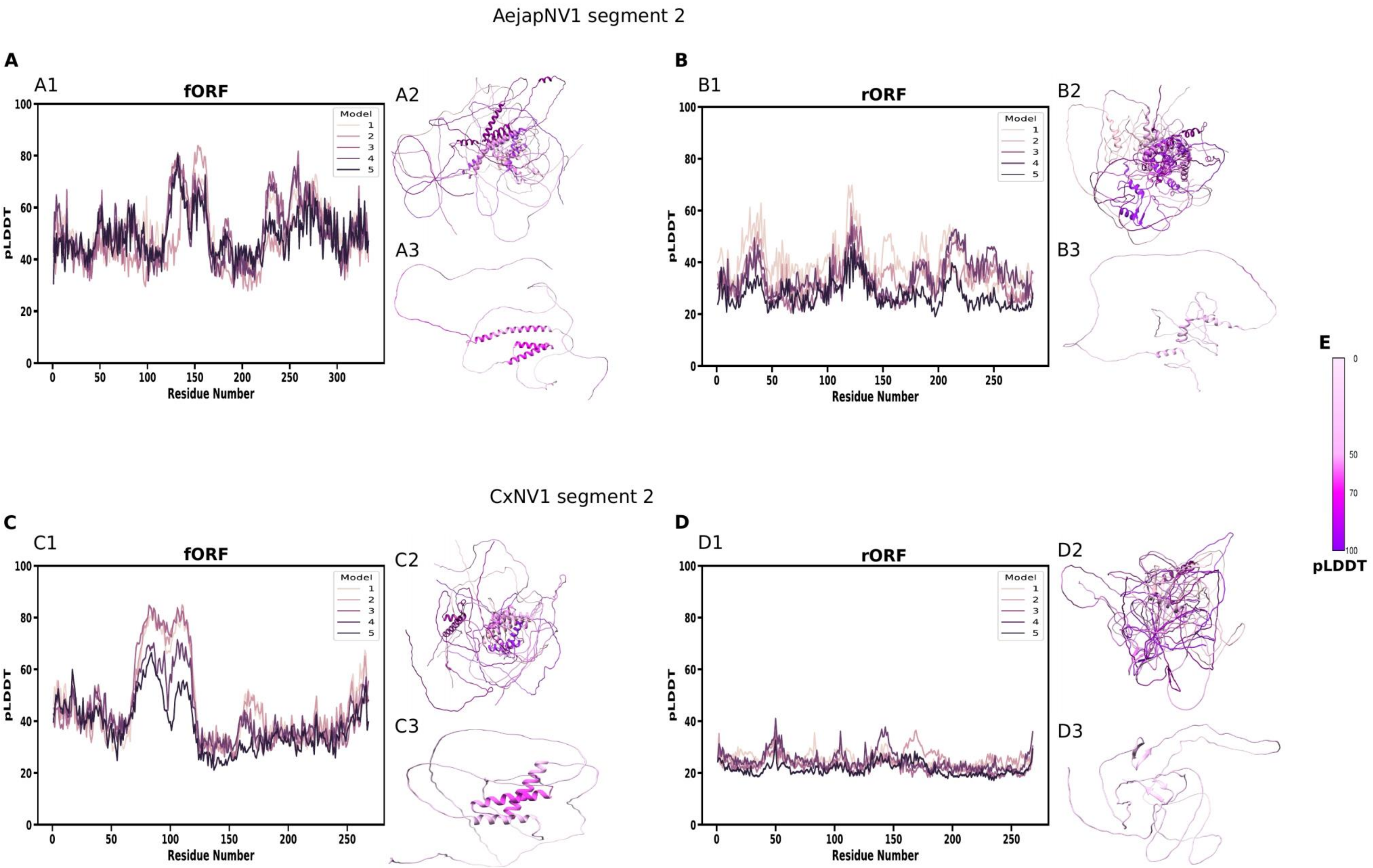

AejapNV1 segment 2
A
A1
fORF
Model
1
2
3
4
5
pLDDT
Residue Number
A2
A3
B
B1
rORF
B2
B3
CxNV1 segment 2
C
C1
fORF
C2
C3
D
D1
rORF
D2
D3
E
pLDDT

Supplementary Figure 6. Tertiary protein structure predictions for segment 2 ORFs of AejapNV1 and CxNV1. AlphaFold per-residue confidence estimation (pLDDT) and calculated models are shown for segment 2 ORFs of AejapNV1 and CxNV1 (GenBank MW226856.1). In **(A)** is shown the AejapNV1 segment 2 fORF, in **(B)** its rORF; in **(C)** the CxNV1 segment 2 fORF and in **(D)** its rORF. A1, B1, C1, and D1 show the pLDDT values of the five calculated models for each ORF. A2, B2, C2, and D2 show the five estimated output models superposed. Confidence ranking values for all models can be found in Suppl. Table 7. A3, B3, C3, and D3 show the model with the highest confidence value for each ORF with pLDDT values rendered in its tertiary structure. The pLDDT color scale is represented in **(E)**. All models were represented in the chart and in the structural superposition using the purple flare color palette, ranging from light purple (model 1) to dark purple (model 5). The pLDDT color scale can be read as: very low confidence (pLDDT < 50), low confidence (70 > pLDDT > 50), confident (90 > pLDDT > 70) and very high confidence (pLDDT > 90).
